# Supplementary material for: ESGO/ESTRO/ESP Guidelines for the management of patients with cervical cancer – Update 2023*
Source: Virchows Arch. 2023 May 5;482(6):935–66. doi: 10.1007/s00428-023-03552-3 (PMC10247855; doi:10.1007/s00428-023-03552-3)
Supplement: Supplementary file 1 — Supplementary file1 This content has been supplied by the author(s). It has not been vetted by BMJ Publishing Group Limited (BMJ) and may not have been peer-reviewed. Any opinions or recommendations discussed are solely those of the author(s) and are not endorsed by BMJ. BMJ disclaims all liability and responsibility arising from any reliance placed on the content. Where the content includes any translated material, BMJ does not warrant the accuracy and reliability of the translations (including but not limited to local regulations, clinical guidelines, terminology, drug names and drug dosages), and is not responsible for any error and/or omissions arising from translation and adaptation or otherwise. (DOCX 793 KB) [file 428_2023_3552_MOESM1_ESM.docx]

**SUPPLEMENTAL DATA - SUMMARY OF EVIDENCE**

**General recommendations**

Given the declining incidence of cervical cancer, centralization is increasingly becoming important to ensure high quality of the diagnostic work-up, treatment, follow-up and rehabilitation. The quality of treatment can be assessed according to the ESGO quality indicators for cervical cancer surgery and radiation therapy published in 2020 and 2023, respectively^1^. European institutions that meet the standards for proper cervical cancer surgery and chemoradiotherapy apply or have already obtained ESGO recognition for cervical cancer surgery and/or radiotherapy build a referral network available on the ESGO website.

The quality indicators include not only a sufficient case load, the training and experience of the surgeon or radiation oncologist, the discussion of each case within a multidisciplinary team, but also the support of continuous recruitment of patients for clinical trials.

Clinical trials have a major impact on cancer care, research practices leading to reduced mortality and prolonged survival, better supportive care and improved understanding of cancer risk, prevention and screening. This research is also leading to the validation of many new cancer treatments such as molecularly targeted therapies and immunotherapies.

**Staging**

**TNM classification & FIGO staging**

The main purpose of cancer staging is to help clinicians predict the prognosis for a cancer patient, to guide treatment planning and follow-up, to evaluate and compare treatment results, to facilitate exchange of information between health professionals and to help in identifying clinical trials that may be appropriate for the patient. The new version 9 of the American Joint Committee on Cancer (9 AJCC TNM) cervical cancer staging aligns with the revised 2018 FIGO (the International Federation of Gynecology and Obstetrics) staging for cervical cancer^2-7^ (see Table 1). The most important changes between FIGO 2009 and 2018 are presented in table 2: 1) the incorporation from imaging and pathologic findings to identify TNM categories and FIGO stage, 2) update of histopathology to reflect human papillomavirus- associated and human papillomavirus- independent carcinomas (WHO 2020), 3) the elimination of horizontal dimension as a parameter for T1a (FIGO IA)^8^, 4) the addition of a subcategory T1b3 to T1b (T1b1 (IB1) ≤2 cm, T1b2 (IB2) >2-≤4 cm, and T1b3 (IB3) >4 cm), 5) introduction of pelvic LN involvement as N1 (FIGO IIIC1), and para-aortic LN involvement as N2 (FIGO IIIC2). Micrometastases (>0.2 mm but ≤2 mm in greatest dimension) are included in Stage IIIC.

It is essential that all cancers must be confirmed by microscopic examination. The histopathologic types are classified as described in the WHO Classification of Female Genital Tumours^9^. Note must be made of LVSI, which does not alter the stage, but may affect the treatment plan^10^. The HPV status of the cancer may be indirectly determined by p16 immunohistochemical overexpression, which is considered a good surrogate marker of HPV-associated tumours or by RNA *in-situ* hybridization. The margins of an excision specimen should be reported to be negative for disease for final staging. Identifying patients suitable for treatment with the immune checkpoint inhibitors (pembrozulimab, nivolumab), may rely on PD-L1 immunoexpression defined as CPS (combined positive score) ≥1^11^.

The FIGO tumour stage is allocated after all imaging and pathology reports are available to generate all 3 categories (TNM) (see Table 1). Pathological findings supersede imaging and clinical findings. Multidisciplinary team discussion of disparate findings is recommended. When in doubt, the lower staging should be assigned. For all morphological subtypes, the term “microinvasive carcinoma” should be avoided and instead the use of specific TNM and FIGO stages is recommended^12^. Stage is not to be altered later, for example at recurrence. A structured checklist is recommended for preoperative imaging to determine staging and other prognostic parameters important for individual treatment. The checklist should include, for example, the largest size of the tumour and, if fertility preservation is desired, the distance from the upper edge of the tumour to the internal cervical os and the craniocaudal length of the cervix; the minimum thickness of the unaffected stroma^13^; invasion of the parametrium; invasion of the vagina (with division of the vagina into upper two-thirds and lower one-third); hydronephrosis (related or unrelated to the extent of the tumour); pelvic side wall invasion into pelvis muscles, fascia, neurovascular structures and skeletal parts of the bony pelvis; bladder/rectal invasion (distinguish between wall and mucosa/lumen invasion); lymphadenopathy (pelvic and/or paraaortic, other areas); adnexal mass(es); other spread (peritoneal spread, visceral organ metastases, etc. ); associated benign conditions; note the presence of anatomical variants; possible tumour-related complications (e.g. thromboembolism, etc.)^14^^,^^15^. It should be noted that the TNM classification more accurately reflects the disease prognosis than the FIGO stage. For example, patients with pelvic and/or para-aortic LN metastases are designated as having stage IIIC regardless of the primary tumour size or local pelvic spread^16-18^. The group of FIGO stage IIIC patients is extremely heterogeneous with highly variable survival rates. FIGO staging should also be documented as both provide complementary information and have been correlated to some extent.

**Prognostic factors**

*Early stage disease*: lymph node involvement appears the most powerful prognostic factor influencing survival rate. The presence of a large tumour, deep stromal invasion (>2/3 of the wall), corpus uteri invasion, and LVSI are other independent factors decreasing survival rates^19-21^. The nodal metastasis rate, paracervical invasion and 5-year cancer death rate are increased with a decrease in the thickness of the remaining intact cervical fibromuscular stroma. The minimum thickness of uninvolved stroma seems to be more useful and objective parameter than the depth of cancer invasion, however, the threshold value of the thickness of cervical fibromuscular stroma sufficient as a barrier against extrauterine spread is not known^22^. Further studies regarding the tumour-free distance and the quadrant of the minimum thickness of the uninvolved cervical stroma should be emphasized^23^. The prognosis of some rare tumours such as neuroendocrine carcinoma of the cervix is worse compared with cervical squamous cell carcinomas or adenocarcinomas. Non HPV-related adenocarcinomas or adenosquamous carcinomas have worse survival than squamous cell carcinomas. Grade has little direct influence on survival within any stage.

*Locally advanced disease*

1) Patient related factors (age, comorbidity, performance status): advancing age is an independent negative prognostic factor for mortality in women with cervical cancer, even after adjusting for race, stage at diagnosis, tumour histology, and treatment^24^. Often this may be due comorbidities and poor performance status, but many times women over 70 are significantly less likely to receive standard of care treatment and much more likely to receive less aggressive (palliative) treatment or no treatment. Diabetes has been associated with poor survival in cervical cancer patients^25^. Thrombocytosis and anemia before treatment and during treatment correlate with worse survival^26^^,^^27^.
2) Factors related to the primary tumour (histologic subtype, tumour size, and degree of invasion into neighboring organs/structures): histological subtype, tumour size and the degree of invasion in the direction of vagina, uterine corpus, parametria (right/left), bladder and rectum as well as (uni- or bilateral) hydronephrosis are well known prognostic factors as also reflected in the FIGO/TNM staging systems. To objectively determine prognosis according to the degree of local tumour spread in all directions, comprehensively and systematically assessed using MRI and clinical examination a tumour score (TS) has been developed, that based on a simple summation of points obtained from the FIGO/TNM staging elements for local tumour spread (see Table 3), and has been shown to precisely predict local control, morbidity and survival following definitive chemoradiotherapy and brachytherapy^28-30^. TS can be used both at diagnosis and during treatment to assess the prognostic impact of local tumour regression observed at brachytherapy, which often is administered after 4-5 weeks of external beam radiotherapy and concomitant chemotherapy.
3) Nodal factors (number, size, morphology and metabolism): The presence of micrometastasis appears to be associated with a negative impact on both the disease-free survival and overall survival and should be treated as macrometastasis^31^. Number of positive LN predict prognosis within stage IIIC1^32^. Position of the nodes may also be important with deteriorating survival with increasing level of nodal involvement: IIIC1 level 1 (small pelvis), IIIC1 level 2 (common iliac) and IIIC2 (para-aortic)^33^.
4) Biomarkers and radiomics (PET-CT hypoxia tracers, DCE-MRI, Doppler imaging; HPV integration; immunomarkers): imaging biomarkers are linked to clinical phenotype, thus with a potential for improving risk stratification and treatment^34^. To predict aggressive phenotype, unchanged, increased or new areas of FDG avidity from baseline signify persistent or progressive disease which is associated with poor survival^35-37^. For prediction of hypoxia novel PET-CT tracers based on fluorine-labeled nitroimidazoles have been tested^38^. Similarly, poorly perfused presumably hypoxic tumours showed low enhancement on dynamic contrast enhanced imaging (DCE-MRI), worse response to chemoradiotherapy, decreased locoregional control and reduced survival^39^^,^^40^. Similarly, low tumour ADC value on diffusion-weighted imaging (DWI) is associated with increased tumour cellularity and predicts an aggressive phenotype^34^. On ultrasound, low vascular indices predict poor treatment response in locally advance cervical cancer^41^. In addition, a hypoxic gene expression classifier was identified^42^.

The growing body of literature indicates the potential of radiomics toward the realization of precision medicine. Imaging features from various sequences (e.g., T2 weighted MRI, DWI-MRI, DCE-MRI) and modalities (e.g. MRI, PET-CT, CT, ultrasound) may be processed separately and then integrated together with clinical, histologic and genomic information toward enhanced discovery of non-invasive biomarkers of prognosis and treatment response.

HPV-associated cervical cancer has a more favorable prognosis than HPV-independent cervical cancer^43^^,^^44^. Approximately 10% of cervical carcinomas are HPV-negative^43^^,^^44^. In a study, patients who had HPV- negative tumours were older, had more advanced disease at diagnosis, and were more frequently diagnosed with non-squamous histology; moreover, they had a significantly worse disease-free survival (60 months vs 132 months) and overall survival (77 months vs 154 months) compared with women who had HPV-positive tumours^44^. Immunomarkers, such as lymphopenia and elevated neutrophil-to-lymphocyte ratio and others have been correlated with worse prognosis in patients with cervical cancer^45^.

5) Predictive factors for specific oncological treatment: with the emerging incorporation of biological therapeutics such as immunotherapy in the treatment of cervical cancer, there is growing need for the establishment of predictive assays for selection of patients to ensure optimal cost/benefit with PD-L1 expression in relation to response to pembrolizumab as an example^46^. Around 35% of cervical squamous cell carcinoma (C-SCC) and 17% of adenocarcinomas expressed PD-L1^47^. PD-L1 overexpression is related to poor overall survival in patients with cervical cancer and poor progression free-survival in Asian patients with cervical cancer^48^. Mismatch Repair Deficiency Microsatellite Instability was found in 11% of cervical cancer^49^. Different biomarkers have been studied to predict response to immune checkpoint inhibitors^50^.

**Local clinical and radiological diagnostic work-up**

The role of pelvic examination is to assess the presence of tumour and perform a tumour biopsy. In addition, evaluation of vagina/vulva/anus is recommended to exclude low genital tract intraepithelial lesions. Clinical examination is insufficient to assess tumour size and rule out parametrial invasion and locally advanced disease^51^. For local staging purposes, MRI or ultrasound performed by trained sonographer provide the highest diagnostic performance, therefore extensive clinical examination using general anesthesia should be omitted^52-55^. CT is inferior to MRI to document local tumour extension, similarly the PET-CT has less predictive value than MRI in terms of detection of local spread because of limited spatial resolution. The implementation of MRI or ultrasound in preoperative workup makes the use of cystoscopy, proctoscopy or intravenous urography redundant. Both imaging modalities can detect the depth of tumour invasion into bladder or rectosigmoid^15^. Biopsy guided by endoscopy might be only required to exclude secondary cancer.

**Nodal/distant diagnostic work-up**

The detection rate of imaging regarding LN and other distant spread depends on their prevalence regarding tumour stage and on size of metastasis. Imaging (ultrasound, MRI, CT or PET-CT) shows high specificity in detection of nodal metastases (>90%) but very low sensitivity in detection of micrometastases (≤2 mm) and small volume metastases (<5 mm). In early stages, the micrometastases are often undetected on imaging and surgical LN assessment is the gold standard for the diagnosis of LN node metastasis(es). T1a1 tumour with no lymphovascular invasion is associated with extremely low incidence of LN metastases and, therefore, LN staging is not useful. In locally advanced cervical cancer, the incidence of extrapelvic disease at the time of initial management is ranging from 10% to 30%, particularly in PALN and/or chest or supraclavicular region. The heterogenous data concerning the diagnostic performance of conventional and functional techniques in nodal staging makes any conclusion regarding the routine diagnostic method unreliable. PET-CT can detect PALN metastases only in patient populations with high probability for metastases. Novel imaging techniques such as WB-DWI/MRI or PET/MRI enable a single examination in locally advanced cervical cancer but remain restricted by limited availability, the need for specialized technical equipment and limited evidence. WB-DWI/MRI can be an option in the staging of pregnant women with cervical cancer. FDG-PET/MRI integrates high-resolution multi-planar morphologic and functional information from MRI with the metabolic data from FDG-PET, which seem to be useful for differentiation between metastatic and benign LN and may reduce false findings. Recent data suggest that FDG-PET/MRI is equivalent to MRI and superior to FDG-PET-CT for local staging of primary tumour; FDG-PET/MRI is comparable to FDG-PET-CT for nodal staging^56^^,^^57^.

Given the limitations of non-invasive techniques to accurately identify small paraortic lymph node (LN) metastasis, the potential role of surgical staging will be discussed in a separate chapter. Inconclusive findings of metastatic lesion should undergo biopsy to confirm or rule out metastatic disease. Tru-cut (core-cut) biopsy is the preferred option because it allows histological assessment of the tumour tissue, fine-needle aspiration biopsy should be avoided.

| **TNM CATEGORY^a^** | **FIGO STAGE^a^** | **CRITERIA** |
| --- | --- | --- |
| **T (TUMOUR)^b^** |  | T CRITERIA |
| TX |  | Primary tumour cannot be assessed |
| T0 |  | No evidence of primary tumour |
| T1 |  | Carcinoma is strictly confined to the cervix (extension to the corpus should be disregarded) |
| T1a^c^ | IA | Carcinoma with maximum depth ≤5 mm |
| T1a1 | IA1 | Measured stromal invasion ≤3 mm in depth |
| T1a2 | IA2 | Measured stromal invasion >3 mm and ≤5 mm in depth |
|  | IB | Carcinoma with deepest invasion >5 mm, limited to the cervix uteri with size measured by maximum tumour diameter. |
| T1b1^d^ | IB1^d^ | Carcinoma with >5 mm depth of stromal invasion and ≤2 cm in greatest dimension. |
|  | IB2 | Carcinoma >2 cm and ≤4 cm in greatest dimension. |
|  | IB3 | Carcinoma >4 cm in greatest dimension. |
| T2 | II | Carcinoma invades beyond the uterus but has not extended onto the lower one-third of the vagina or to the pelvic wall |
| T2a | IIA | Involvement limited to the upper two-thirds of the vagina without parametrial invasion |
| T2a1 | IIA1 | Carcinoma ≤4 cm in greatest dimension |
| T2a2 | IIA2 | Carcinoma >4 cm in greatest dimension |
| T2b | IIB | With parametrial invasion but not up to the pelvic wall |
| T3^e^ | III^e^ | Carcinoma involves the lower third of the vagina and/or extends to the pelvic wall and/or causes hydronephrosis or non-functioning kidney |
| T3a | IIIA | Carcinoma involves the lower third of the vagina, with no extension to the pelvic wall. |
| T3b | IIIB | Extension to the pelvic wall and/or hydronephrosis or non-functioning kidney (unless known to be due to another cause). |
| T4^f^ | IVa^f^ | Carcinoma has involved (biopsy-proven) the mucosa of the bladder or rectum or has spread to adjacent organs. |
| **N (NODE) CATEGORY^g^** |  |  |
| Nx |  | Regional lymph nodes cannot be assessed |
| N0 |  | No regional lymph node metastasis |
| N0(i+)**^h^** |  | Isolated tumour cells in regional lymph node(s) ≤0.2 mm or single cells or clusers of cells ≤200 cells in a single lymph node cross-section |
| N1 | IIIC | Regional lymph node metastasis to pelvic lymph nodes only |
| N1mi**^i^** |  | Regional lymph node metastasis (>0.2 mm but ≤2.0 in greatest dimension) to pelvic lymph nodes |
| N1a |  | Regional lymph node metastasis (> 2 mm in greatest dimension) to pelvic lymph nodes |
| N2 |  | Regional lymph node metastasis to para-aortic lymph nodes, with or without positive pelvic lymph nodes. |
| N2mi**^i^** |  | Regional lymph node metastasis (>0.2 mm but ≤2.0 in greatest dimension) to para-aortic lymph nodes, with or without positive pelvic lymph nodes |
| N2a | IIIC2 | Regional lymph node metastasis (> 2.0 in greatest dimension) to para-aortic lymph nodes, with or without positive pelvic lymph nodes |
| **M (METASTASIS) CATEGORY** |  |  |
| M0 |  | No distant metastasis |
| cM1^j^ | IVB | Distant metastasis (clinical category) |
| pM1^j^ | IVB | Distant metastasis (pathologic category) |
|  |  |  |
| ^a^All imaging modalities and pathology can be used, when available, to supplement clinical findings with respect to tumour size and extent, in all stages. Pathological findings supercede imaging and clinical findings; ^b^Involvement of lymphovascular spaces should not change the staging, but may affect the treatment plan; ^c^The diagnosis of T1a1,2 is made on microscopic examination of a surgical specimen, which includes the entire lesion. The depth of invasion should not be greater than 3 or 5 mm, respectively, from the base of the epithelium. For T1a1,2 the horizontal dimension is no longer considered in defining the upper boundary of a T1a carcinoma^2^. The margins of a cone specimen should be reported to be negative for disease to do the final pathological stage. If the margins of the cone biopsy are positive for invasive cancer, the patient is assigned to T1b1. Exceptions are allowed, for example, for large exophytic tumours>2 cm in the largest dimension, which should be staged based on the largest dimension even if they are superficially invasive (≤5 mm)^58^. In some situations (e.g. in ulcerated tumours) it is not possible to measure the depth of invasion. In such cases, the tumour thickness may be measured, and this should be clearly stated in the pathology report together with the reasons why the thickness and not the depth of invasion is given. In such cases, the pathologist and clinician should correlate tumour thickness with depth of invasion for staging and management purposes^58^; ^d^A new primary tumour size cutoff value of 2 cm enables to evaluate potential candidates for fertility-sparing treatment. For this purpose, craniocaudal cervical length, tumour-to-internal cervical os distance are also measured; ^e^The pelvic wall is defined as the muscle, fascia, neurovascular structures, and skeletal portions of the bony pelvis; ^f^Bullous edema does not permit a case to be assigned to stage IVA; ^g^Adding notation of r (radiology) and p (pathology) to indicate the findings that are used to allocate the case to Stage IIIC (e.g. IIICp, IIICr). The type of pathology technique used should also be documented. The suffix (f) is added to the N category when metastasis is identified by fine-needle aspiration or core biopsy. The suffix (sn) is added to the N category when metastasis is identified only by sentinely lymph node biopsy. When in doubt, the lower staging should be assigned; **^h^**Isolated tumour cells do not change the stage but their presence should be recorded;**^i^**Micrometastases are included in Stage IIIC. Isolated tumour cells do not change the stage but their presence should be recorded; ^j^Includes metastasis to inguinal, mediastinal, supraclavicular and other lymph nodes regions beyond abdomen, intraperitoneal disease, lung, liver, or bone, excludes metastasis to pelvic or para-aortic lymph nodes or vagina. | | |

**Table 1 - Revised FIGO 2018 and TNM staging cervical cancer****^2^****^,^****^6^**

| **FIGO 2009** | **FIGO 2018** |
| --- | --- |
| Staging was based primarily on clinical findings, in addition plain radiographs; including intravenous pyelography can be used for staging. | All imaging modalities such as ultrasound, CT, MRI or PET-CT and pathologic findings can be used to complement clinical evaluation in assessing both tumour size and extent. The method used to assign the stage should be recorded. Pathologic findings take precedence overclinical assessment and imaging findings in assigning the FIGO stage. |
| **Stage IA** | **Stage IA** |
| The classification of IA stage depended on both the extent of horizontal spread and the depth of disease invasion. | The horizontal dimension of the lesion is no longer considered in defining the upper boundary of IA stage. |
| IA carcinoma with maximum depth of invasion ≤5 mm with a horizontal spread 7.0 mm of less. | IA Carcinoma with maximum depth of invasion ≤5 mm. |
| **Stage IB** | **Stage IB** |
|  | The creation of new subcategory (IB3) enables more consistent contribution of tumour size to prognosis. In addition, tumour size cutoff value of 2 cm enables to evaluate potential candidates for fertility-sparing treatment. |
| IB1 Clinically visible lesion ≤4.0 cm in greatest dimension. | IB1 Invasive carcinoma with >5 mm depth of stromal invasion and ≤2 cm in greatest dimension. |
| IB2 Clinically visible lesion >4.0 cm in greatest dimension. | IB2 Invasive carcinoma >2 cm and ≤4 cm in greatest dimension. |
|  | IB3 Invasive carcinoma >4 cm in greatest dimension. |
| **Stage III** | **Stage III** |
| The tumour extends to the pelvic wall and/or involves lower third of the vagina and/or causes hydronephrosis or non-functioning kidney. The lymph node status is not incorporated in stage III. | The carcinoma involves the lower third of the vagina and/or extends to the pelvic wall and/or causes hydronephrosis or non-functioning kidney and/or involves pelvic and/or PALN. |
| IIIA Tumour involves lower third of the vagina, with no extension to the pelvic wall. | IIIA The carcinoma involves the lower third of the vagina, with no extension to the pelvic wall. |
| IIIB Extension to the pelvic wall and/or hydronephrosis or non-functioning kidney. | IIIB Extension to the pelvic wall and/or hydronephrosis or non-functioning kidney (unless known to be due to another cause). |
|  | IIIC Involvement of pelvic and/or PALN (including micrometastasis), irrespective of tumour size and extent |
|  | IIIC1 Pelvic lymph node metastasis only. |
|  | IIIC2 PALN metastasis. |

**Table 2 - Key updates of the 2018 FIGO classification compared to the prior version**

**Locations included in TS score**

1. Maximal tumour diameter at the cervix (mm)
2. Left parametrium: not involved, proximal, distal, pelvic wall
3. Right parametrium: not involved, proximal, distal, pelvic wall
4. Vagina: not involved, upper 1/3, middle 1/3, lower 1/3
5. Corpus uteri: not involved, lower 1/3, middle 1/3, upper 1/3
6. Bladder: not involved, bladder wall, bullous edema, mucosa involvement
7. Ureter: Not involved, unilateral hydronephrosis, bilateral hydronephrosis
8. Rectum: Not involved, mesorectum, rectal wall, mucosa

**Table 3 - Locations included in TS score**

**Management of T1a disease**

Series published since the 2018 ESGO/ESTRO/ESP guidelines have led to minor changes in the 2022 update^59-88^. Sentinel node procedures are now preferred to systematic node dissections in patients for whom nodal staging is considered necessary. The 2022 update includes a new recommendation for SH for patients with T1a1 or T1a2 adenocarcinoma who have completed childbearing^80^^,^^88-90^.

Diagnosis and management of T1a cervical cancer should be based on expert pathology of an intact histologic specimen (cone or excsional specimen). Loop or laser conization is preferable to cold knife conization in women desiring preservation of fertility. Fragmentation of the specimen and thermal artefacts should be avoided as they can obscure the extent of the tumour and margin status. The specimen should be oriented for the pathologist to determine the exact location of the tumour. The pathology report should specify depth of invasion, the status of the margins, and assessment of LVSI; the horizontal extent is optional since it is no longer included in TNM/FIGO staging system. The status of the margins is important because patients with positive margins aftera LEEP or cold knife conization have a higher frequency of residual disease in completion hysterectomy specimens than those with clear margins.

The recommendations for treatment of stage IA have been updated in that a sentinel node procedure (not a systematic dissection) is recommended for LN staging if nodal staging is to be done^80^^,^^88^. LN staging is not indicated in T1a1 LVSI-negative patients but can be considered in T1a1 LVSI-positive patients (unchanged recommendation).

**Management of T1b1, T1b2, and T2a1 tumours**

**Laparotomy is the standard surgical approach to parametrectomy**

The first version of the ESGO-ESTRO-ESP guidelines had been published just before the LACC study presentation in 2018, so it recommended minimally invasive surgery (MIS) as the preferred approach for radical hysterectomy^91^. This new version, based on new evidence, recommends laparotomy as the standard approach for all procedures which include radical parametrectomy. However, it leaves space for MIS for LN staging, thus allowing sentinel lymph node (SLN) biopsy to be performed by laparoscopy or robotic surgery, and if the frozen section result is negative, only then laparotomy is performed. The radical uterine procedure can therefore be done from a transverse incision. Moreover, the new guidelines even open up an option to perform radical hysterectomy by MIS in a very low-risk cohort of patients with small tumours after conization with free margins. A retrospective multicentre study did not find an increased risk associated with MIS approach in such a low-risk cohort, and a post hoc analysis of the LACC study has cometo the similar conclusion in patients after fertility sparing treatment^92^^,^^93^.

**Sentinel lymph node biopsy strongly recommended in primary surgical management**

The new guidelines unequivocally recommend performing SLN in all patients with early-stage disease as part of primary surgical management. It reflects the accumulating data on much higher detection rate of LN positivity thanks to pathologic ultrastaging of SLN. In the recent prospective Sentix study, almost 60% of cases with micro or macrometastases in SLN were detected only by pathological ultrastaging^94^.

**Random pelvic lymph nodes not recommended for intraoperative assessment**

Intraoperative pathological assessment of SLN allows the detection of only about half of the cases with N, but in this group it allows preventing the combination of two radical treatment modalities. Such algorithm was also used in the SENTIX prospective study, in which all SLN were intraoperatively processed by frozen section and radical hysterectomy was abandoned in case of metastatic involvement. In 8% of patients, uterine radical procedure was abandoned intraoperatively, and only 7% was referred to adjuvant radiotherapy after radical surgery due to N1 status from the final pathology^94^. The updated version of the guidelines keeps recommending the intraoperative assessment of all SLN and/or suspected pelvic LN. However, the recommendation to examine randomly selected pelvic lymph nodes if SLN were not detected, has been omitted, because no method is available to determine which pelvic LN should be selected and a larger number of pelvic LN cannot be assessed by frozen section.

**Systematic pelvic lymphadenectomy remained a standard procedure for lymph node staging**

Although a large number of retrospective and several prospective studies have demonstrated the high sensitivity of SLN ultrastaging for pelvic LN status, there is currently only one prospective study in which SLN biopsy was not followed by a systematic pelvic lymphadenectomy^95^. Due to the lack of prospective evidence on the safety of avoidance of systematic procedure, pelvic lymphadenectomy remains recommended staging procedure after SLN biopsy in T1b/T2a disease. Only the results of the ongoing 3 prospective studies may change clinical practice in the future^96-98^.

**Limitation of lymph node dissection to the anatomical level I if sentinel lymph node not detected**

In the early stages, the distant spread of the disease is almost exclusively via lymphatic channels, and anatomically it almost always preserves the sequence of first pelvic and only later para-aortic LN. This is also why the staging of pelvic LN in cervical cancer is such an important prognostic parameter. In the SENTIX study, which is currently the largest prospective study with SLN biopsy and ultrastaging, such an anatomical gradient was demonstrated even within the pelvic lymph nodes in the cohort of 355 patients^94^. Only in 2% of patients an isolated positive SLN was detected at the pelvic level II cranially to iliac bifurcation, i.e., in common iliac or presacral regions. Therefore, the updated guidelines recommend limiting the LN dissection to the pelvic level I, below the iliac vessel bifurcation, if SLN is negative on frozen section bilaterally in the pelvis.

**Precise criteria for selection of candidates for ovarian preservation and transposition**

In the updated guidelines, the conditions for ovarian preservation are specified more precisely. Currently, data on the safety of ovarian preservation are available only from retrospective studies, and mostly not as a risk of recurrence in the ovaries, but as microscopic ovarian metastatic involvement from salpingo-oophorectomy specimen^99-101^. Based on limited evidence, ovarian preservation can only be considered in women with usual tumour types. If the ovaries are preserved, a salpingectomy should always be performed.

**Avoidance of radical surgery if lymph node involvement is detected intraoperatively**

In the updated guidelines, the recommendation to abandon further radical surgery, both pelvic lymphadenectomy and radical hysterectomy, if any LN involvement is detected intraoperatively is retained. This recommendation was rated as controversial in the ESGO survey conducted in 2021 (data not published). However, another supporting argument for such a management appeared since the original guidelines were published. An ABRAX international retrospective study was designed to tackle this controversy, including only cases in which LN involvement was detected intraoperatively. Not only the completion of radical hysterectomy did not improve survival in the whole cohort, but no signal towards survival benefit was found in any of the subgroups, regardless of the tumour size or tumour type^102^.

**Preoperative brachytherapy**

A multimodal strategy combing preoperative uterovaginal brachytherapy and radical hysterectomy performed 6 to 8 weeks later has been proposed in a limited number of centres for patients with T1b1 and T1b2 tumours (LN negative), with a level of evidence mainly based on retrospective series. It aims at eradicating local risk factors and avoiding adjuvant radiotherapy in patients with intermediate risk factors^103^^,^^104^. High rate of pathological complete response (>70%) and excellent outcome were reported, as shown in a recent meta-analysis of 3 randomized controlled trials and 10 non-randomized studies^105^. A high-quality randomized controlled study is however required to confirm the benefit of this strategy and for now the use of preoperative brachytherapy followed by surgery (type A) is an option only in teams experienced in this approach (unchanged recommendation).

**Fertility sparing treatment**

Many studies addressing the fertility sparing treatment, mainly retrospective series, have been published in the course of the past 5 years, confirming the validity of the original ESGO/ESTRO/ESP guidelines^60^^,^^66^^,^^72-75^^,^^83^^,^^84^^,^^106-155^. Given the possible spectrum of fertility sparing treatment, patient’s councelling and treatment should only be done in centres, which can offer all kinds of fertility sparing treatment (sentinel and full pelvic lymphadenectomy, conization, simple trachelectomy, radical trachelectomy, fertility sparing treatment following NACT, comprehensive staging in patients with necessity to abandone fertility sparing treatment) and perform an adequate number of procedures annnually^142^. Indeed, there is a clear tendency towards less radical cervical surgery, but upcoming data are not uniform^145^^,^^156-161^. The psychological aspects of fertility sparing treatment should gain more attention. Patients should be very extensively counselled about potentially more aggressive behavior of neuroendocrine carcinomas, HPV-independent adenocarcinomas and carcinosarcomas, and high uncertainties with respect to fertility sparing treatment. The fertility sparing treatment of rhabdomyosarcoma has been addressed as part of the ESGO-ESTRO-SIOPe guidelines for the multidisciplinary management of vaginal cancer (<https://guidelines.esgo.org/>).

Any pregnancy following fertility sparing surgical procedure is associated with increased risk of PROM, preterm delivery, longer neonatal intensive care stay and lower birth weight, at least for conization and highest for abdominal radical trachelectomy^157^^,^^162-164^. Various preventive methods can be discussed with the patient (e.g. regular measurement of vaginal pH- value, laparoscopic placement of cerclage, bed-rest and abstain from sexual intercourse, sick note, vaginal progesteron application, screening and treatment of asymptomatic bacteriuria, screening for cervical incompetence and progressive cervical shortening by transvaginal ultrasonography, fetal fibronectin testing, screening (and treatment) for asymptomatic vaginal infection, total cervical closure according to Saling and cervical cerclage, etc.)^165^. Patients should be given full details related to the different techniques of radical trachelectomy. Different surgical approaches for radical trachelectomy have been described such as radical vaginal trachelectomy, abdominal radical trachelectomy, total laparoscopic radical trachelectomy and robotic-assisted laparoscopic radical trachelectomy^66^^,^^75^^,^^83^^,^^84^^,^^108^^,^^112-114^^,^^116^^,^^117^^,^^119-123^^,^^126^^,^^129^^,^^131^^,^^135-138^^,^^140^^,^^143^^,^^146^^,^^147^^,^^154^. To date, no randomized trial comparing these approaches has been published. Differences in oncologic outcome, pregnancy rates as well as pre-term delivery rates have been reported^157^^,^^166^^,^^167^. Total laparoscopic radical trachelectomy should be used with caution based on the results of LACC and IRTA trial. Besides promising oncologic results, abdominal radical trachelectomy is associated with lowest pregnancy rate. Most comprehensive data exist for radical vaginal trachelectomy (if performed in a highly specialized centre). Moreover, radical vaginal trachelectomy appears to have the best oncological and pregnancy outcome. Robotic-assisted laparoscopic radical trachelectomy potentially might be an alternative to radical vaginal trachelectomy, but more and longer follow-up data are needed. Additional removal of upper parauterine lymphovascular tissue, if detectable, has the potential to improve validity of sentinel concept, but further studies are needed^168^. Permanent cerclage should be placed in all patients during simple or radical trachelectomy. However, best material for permanent cerclage is still undecided. Encapsulation of residual tumour with adequate vaginal cuff at initial step of the surgery and avoidance of uterine manipulator use seems to be advantageous. No imaging modality can exactly predict the extent of requested local resection in order to reach tumour free margins with adequate safety distance. Postoperative histologic proven tumour free resection is mandatory, but indispensable safety margins are still a matter of debate.

NACT followed by fertility-sparing surgery is a promising strategy that might allow fertility preservation in patients with tumours >2 cm while providing acceptable oncologic outcome^106^^,^^130^^,^^145^^,^^150^^,^^155^. However, the optimal number of chemotherapy cycles, chemotherapy regimen as well as extent of cervical resection following NACT, are yet to be defined. Another, also experimental, option for patients with tumours >2 cm is upfront abdominal radical trachelectomy. Given that published series are limited in size, there are data demonstrating that neo-adjuvant chemotherapy followed by radical vaginal or abdominal trachelectomy has similar oncological results as assisted reproductive technology alone, but induce better pregnancy rate. Therefore, first mentioned approach should be the preferred one and should be performed in reference centres with a prospective evaluation of patients.

No recommendation can be provided for earliest possible realization of childbearing following fertility sparing treatment. After finishing of wound healing pregnancy seems possible, also use of *in vitro* fertilisation techniques. Routine hysterectomy following fertility sparing treatment and finished family planning is not generally recommended because it does not seem to increase oncologic safety despite limited available data.

Secondary hysterectomy should only be considered in patients with persistent clinical symptoms such as dysmenorrhoea, dyspareunia, vaginal discharge, irregular bleeding or repeated cevical stenosis. Repeated abnormal Pap test after fertility sparing treatment is frequently observed with lower clinical relevance. Patient’s strong wish can be another reason for considering secondary hysterectomy.

**Invasive cervical cancer diagnosed on a SH specimen**

Pathological examination of a hysterectomy specimen occasionally reveals invasive cervical cancer. This may be observed in case of hysterectomy for benign condition of the uterine corpus, or prolapse, or for management of preinvasive cervical disease. This clinical situation raises specific management issues: SLN no longer applicable, frequent need of repeated therapeutic interventions, difficult planning of radiation therapy - with in addition presence of bowel in the middle of the pelvis and possible adhesions, risks associated with cut-through surgery. It is in most circumstances the result of improper or misinterpreted preoperative workup. The attention of general gynecologists must be drawn on the need to carefully rule out cervical cancer before any decision of SH. It is mandatory to investigate by imaging and/or endocervical biopsy any enlarged cervix, and not to overlook abnormal uterine bleeding in circumstances when the presence of metrorrhagia is not an unusual symptom, for example in case of uterine leiomyoma or genital prolapse, or intraepithelial cervical disease. In the latter case, care must be taken to make sure that the biopsies have been performed in the most abnormal area of the cervix. Cone biopsy should be considered in case of doubt.

The recommendations are derived from the ones applying to the general case, with the objective of avoiding any discrepancy compared to general recommendations. The rationale when elaborating the recommendations specific to postoperative finding is to adapt the management, taking into account the circumstance, in order to meet the same objectives. Workup including imaging, need of pathology review and tumour board, surgical staging of nodal disease when applicable, availability of both surgery and radiation therapy in most cases, and avoidance of combining both modalities if possible, are principles shared with the general case. Based on these principles, the algorithm of staging procedures (imaging with or without surgery), surgical management, or radiation therapy according to clinical and pathological stage should also be as consistent as possible with the general situation. In this specific situation, additional available decisional parameters are the pathological margin status and presence of residual tumour.

The recent literature has not added a lot to knowledge^169^^,^^170^. The most informative recent publication on the outcome of management modalities is a paper derived from the SEER database^170^. The authors found that postoperative radiation therapy appears effective, however possibly less than radical surgery. The choice between surgery and radiation should however take into account the technical difficulties of surgery, the risk of postoperative complication, and the risk to have to offer postoperative radiation therapy. Surgery performed in the absence of the uterus follows the same principles than radical hysterectomy and consists in removing the vaginal cuff and adjacent paracervix. Planning of radiation must also be adapted to the absence of uterus and possible bowel adhesions.

**Management of locally advanced cervical cancer (T1b3-T4a)**

**CTRT and BT**

The worldwide clinical implementation of image guided EBRT and IGABT (MRI) has provided a major breakthrough in the treatment of locally advanced cervical cancer with a significantly improved loco-regional control and a reduced morbidity in all stages of locally advanced cervical cancer compared to previous benchmark studies^171^^,^^172^.

**Combining EBRT and BT**

IGBT encompassing combined intracavitary/interstitial techniques is pivotal for obtaining local control and provides superior survival compared with patients where BT is replaced with a boost of EBRT^171^^,^^173-175^. The optimal combination of EBRT and BT involves an initial 5 weeks of EBRT with concomitant cisplatin providing not only tumour regression but also a homogenous dose platform from which an adaptive boost to above 90 Gy is delivered to the remaining tumour and the cervix (high-risk clinical target volume) at the time of BT^172^. The BT boost should be administered in the final weeks of an overall treatment time (EBRT + BT) of maximally 50 days^171^. The optimal dose of EBRT has been shown to be whole pelvic 45 Gy in 25 fractions^176^. Parametrial boost by EBRT should be not be used as substantial dose heterogeneity from EBRT makes summation of doses from EBRT and BT unsafe and incurs unnecessary dose to nearby organs at risk^177^. Pathological nodes should be boosted to about 60 Gy (EQD2) with a simultaneous integrated boost considering the expected dose contribution from EBRT^33^^,^^172^^,^^178-180^.

**Persistent tumour following CTRT and BT**

Persistence of the primary tumour following definitive CTRT and BT may be difficult to diagnose on imaging even by use of MRI and PET-CT^181^^,^^182^. Further, many patients with apparent persistent disease on imaging at 3 months after treatment achieves complete remission within 6-9 months of further follow-up without further treatment^175^. Repetitive imaging and biopsies may therefore be needed to establish the diagnosis of true persistent local disease. Circulating cell-free HPV DNA in plasma is currently being investigated as a new tool for assessing treatment response and monitoring of the disease^183^.

**NACT before definitive CTRT and BT**

NACT given before definitive CTRT and BT has so far failed to show any benefit^184^^,^^185^. A major reason for these findings is likely the prolongation of the overall treatment time including the NACT, which may induce accelerated repopulation.

**Adjuvant chemotherapy following definitive CTRT and BT**

A randomized phase III study reported more than 10 years ago showed that the addition of two courses of adjuvant gemcitabine/cisplatin improved progression free survival^186^. However, this study raised several questions and has not seen widespread implementation^187^. Other randomized trials including the notable OUTBACK trial as well as a meta-analysis, have unfortunately been negative^188-190^.

**Completion/adjuvant hysterectomy in the context of definitive CTRT and BT**

Completion of radical hysterectomy does not improve survival of patients with intraoperatively detected LN involvement which any way are referred for definitive CTRT and BT^102^. Likewise, studies on adjuvant hysterectomy following primary radiotherapy and BT have been negative^191-195^. In addition, substantial perioperative and postoperative late morbidity has been observed^192^^,^^194^.

**NACT before surgery**

Randomized studies conducted before the advent of concomitant chemotherapy showed a survival benefit for NACT and surgery compared with definitive radiotherapy^196^. However, 2 recent randomized trials employing CTRT did not show this benefit^197^^,^^198^. A meta-analysis of the data from these two studies has even suggested a 32% higher risk of relapse with the neoadjuvant strategy^199^. Irrespective of the types of chemotherapy used over the last 30 years, the proportion of patients remaining inoperable after NACT has been stable at 25-30%. The proportion of the patients being referred for postoperative adjuvant radiation or chemoradiotherapy following NACT and surgery and thus exposed to triple treatment has also been stable at 25-30%^196^.

**Role of surgery in T1b3 and T2a2 (LN negative) tumours**

The management of patients with IB tumours and tumour size greater than 4 cm remains controversial. That is why a separate chapter was designated for this topic in the updated guidelines. Currently, there is no evidence demonstrating the superiority of either surgical treatment or primary chemoradiotherapy in this cohort of patients. Prospective studies mostly compared primary radiotherapy with chemoradiotherapy, and in an older Italian study, survival did not differ between the two types of management^200-202^. Inevitably, all these patients meet the criteria for intermediate-risk group (LN negative but a combination of negative prognostic factors such as larger tumour size, LVSI and deep stromal invasion), just by combining the size of the tumour with the depth of invasion. Surgical treatment should therefore only be used if radical hysterectomy remains the only main treatment modality without adjuvant treatment. In recent years, several retrospective studies have shown no survival benefit of the adjuvant treatment after radical hysterectomy in patients with intermediate risk tumours^203-206^. According to the updated guidelines, surgery in these patients should be limited to highly specialized centres with experience with type C radical hysterectomy.

**Recurrent/metastatic disease**

The phase 3 trial, GOG#240 analyzed the addition of bevacizumab, anti-VEGF agent, to the standard of care at that stage, platinum-doublet chemotherapy^207^. The introduction of the antiangiogenic agent bevacizumab has extended median overall survival from about 12 to 17 months, since becoming the standard of care for this population. The addition of bevacizumab to platinum-based chemotherapy led to an unprecedented improvement in median overall survival for those patients with recurrent/metastatic disease, however, a new specific adverse event linked to bevacizumab use appeared, fistula. At final analysis the overall incidence of fistula (Grade 2 and Grade 3) was 8.6% among patients treated with bevacizumab compared with 1.4% for those without. All patients who developed fistula had had prior radiotherapy. No fistulas resulted in surgical emergencies, sepsis, or death, and in addition to pelvic irradiation, other factors associated with fistula included pelvic disease, pre-existing hypertension, and current tobacco use^207^.

MK-3475-826/KEYNOTE-826 is a phase III randomized, double-blind, placebo-controlled study, designed to assess the benefit of adding pembrolizumab to chemotherapy with or without bevacizumab, in persistent, recurrent, or metastatic cervical cancer patients, in the frontline setting^208^. A total of 617 eligible patients were randomly assigned in a 1:1 ratio to receive pembrolizumab/placebo plus platinum-based chemotherapy for up to 6 cycles and bevacizumab at the investigators’ discretion. The dual primary endpoints were progression-free survival and overall survival, each tested sequentially in patients with a PD-L1 CPS ≥1, in the intention-to-treat population, and finally, in patients with a PD-L1 CPS ≥ 10. After a median follow-up of 22.0 months the overall survival at 24 months was 53.0% in the pembrolizumab group and 41.7% in the placebo group (HR 0.64; 95% CI, 0.50 - 0.81; p<0.001), 50.4% and 40.4% (HR 0.67; 95% CI, 0.54 - 0.84; p<0.001), and 54.4% and 44.6% (HR 0.61; 95% CI, 0.44 - 0.84; p=0.001), in the PD-L1 CPS ≥1, intention-to-treat, and PD-L1 CPS ≥10 populations, respectively. Regarding the protocol-specified subgroup analysis, the overall survival benefit provided by the addition of pembrolizumab was generally consistent across all patient subgroups. However, PD-L1 CPS<1 subgroup did not seem to obtain survival benefit among the PD-L1-selected subpopulations. Despite that the trial met its primary end-point in the intent to treat population, based on the aforementioned subgroup analysis; both FDA and EMA have recently approved the use of pembrolizumab added to platinum-based chemotherapy plus or minus bevacizumab only for those patients whose tumours are CPS ≥1.

The phase III trial EMPOWER-Cervical-1/GOG-3061/ENGOT-cx9 compared cemiplimab, an anti-PD-1 antibody, versus the investigator’s choice of single-agent chemotherapy in patients with advanced cervical cancer who had progressed after first-line platinum-containing chemotherapy^209^. It is important to underscore the patients were included regardless of PD-L1 expression status. The primary endpoint was overall survival which was analyzed hierarchically in patients with squamous cell carcinoma followed by the intention-to-treat population. The trial was stopped, after the second planned interim analysis, based on pre-specified criteria for efficacy in the squamous-cell carcinoma populationthat demonstrated significantly improved overall survival in patients receiving cemiplimab monotherapy. Per-protocol final survival analysis was performed after 363 overall survival events were observed in the squamous-cell carcinoma patients’ cohort, at a median follow-up of 30 months. These outcomes were recently presented at the 2022 ESMO congress. In the squamous cell carcinoma population, median overall survival was significantly longer with cemiplimab than with chemotherapy (10.9 months versus 8.8 months; HR 0.69; 95% CI, 0.56 - 0.85; p=0.0023), as well as in the overall population (11.7 months versus 8.5 months; HR, 0.65; 95% CI, 0.54 - 0.79; p<0.001). Moreover, overall survival was evaluated according to the status of PD-L1 in an exploratory analysis. In the most recent update, of 608 randomized patients, only 371 (61%) had valid baseline PD-L1 samples (182 in the cemiplimab arm and 189 in the chemotherapy arm). In the PD-L1 tested population, cemiplimab increased overall survival versus chemotherapy in patients with both PD-L1 ≥1% (HR 0.61; 95%CI, 0.45 to 0.83) and PD-L1<1% (HR 0.65; 95%CI, 0.42 - 0.98), with 38% and 35% lower risk of death, respectively. Following the final overall survival results of this trial, on 13^th^October 2022, the European Committee for Medicinal Products for Human Use adopted a positive opinion for cemiplimab in the treatment of patients with recurrent or metastatic cervical cancer, regardless of PD-L1 status, and disease progression on or after platinum-based chemotherapy.

Chemotherapy is the standard treatment in stage IVB cervical cancer. However, given that many women have a significant pelvic disease burden. Due to this fact, several retrospectives’ series have studied the role of pelvic radiation in addition to chemotherapy for primary treatment^210^^,^^211^. The conclusion of all these series is that pelvic radiation in addition to chemotherapy gives a significant overall survival benefit.

**Follow-up during and after treatment/long-term survivorship**

Cancer survivors include those who start treatment, continue treatment, have completed treatment, or are in clinical remission. Their follow-up focuses on assessing the effect of treatment and detecting recurrence, preventing and screening for subsequent primary tumours (oncological follow-up), but also on preventing, diagnosing and treating common sequelae of cancer and cancer treatment (monitoring quality of life and side-effects). Follow-up should be performed and coordinated by a physician experienced in the treatment and follow-up of gynaecological cancer patients. Communication with all physicians involved in survivorship care, including primary care physicians (i.e., general practitioners), is essential.

It is recommended to provide survivors with a summary of information about their cancer history, including their treatment, side effects, and recommendations for follow-up, health promotion and prevention (in a survivorship care plan). At the time of transition of follow-up from a specialized centre to the primary care physician (or gynaecologist) such a long-term survivorship care plan provides an opportunity to transmit important information to the patient and relevant health care providers about long-term follow-up and potential late effects of cancer treatment^212^.

**Oncological follow-up**

There is no evidence on the most appropriate follow-up strategy to detect tumour recurrence and prospective studies focusing on new follow-up strategies are warranted. The risk of recurrence is individual and depends on prognostic factors, treatment modality and patient characteristics. Therefore, surveillance programmes for patients undergoing treatment and after treatment for cervical cancer shoud be individualized to take these aspects into account.

The starting point for follow-up should be a treatment evaluation with documentation of tumour response. Recommended imaging after chemoradiotherapy is pelvic MRI, including DWI (diffusion-weighted imaging) (local extent) and CT or PET-CT (extrapelvic spread)^55^. The implementation of PET/MRI is still limited due to low availability, high costs and the need for specialized technical expertise.

Typically, more than three-fourths of recurrences occur within 2-3 years after primary treatment, follow-up should be more intensive during this period^213^^,^^214^. The most frequent recurrence sites are the pelvis (vaginal vault, cervix, parametrium, and pelvic wall) and the paraaortic LN. Patients without relapse rarely need to be followed in a specialized centre for more than 5 years after primary treatment. Counseling patients about the signs of recurrence remains an important part of survivorship care. Follow-up visits should include, at a minimum, a complete physical examination, including pelvic examination, and a patient history. Vaginal vault cytology has a low positive predictive value for detecting recurrence after chemoradiotherapy and surgery and is therefore not routinely recommended^213^^,^^215^. Instead of vaginal cytology, HPV testing may be useful in identifying vaginal precancer lesions or recurrence, but robust evidence is still lacking^215^. Imaging and laboratory tests, including serum biomarkers, are not routinely recommended because there is no convincing evidence that earlier detection of recurrence is associated with improved survival in recurrent cervical cancer.

Intensified oncological follow-up after treatment should focus on a group of patients with potentially recurrent disease that can be treated with curative intent or whose treatment will lead to long-term survival (i.e., those with early diagnosis of locoregional recurrence). This group should be offered more intensive follow-up with imaging and biomarkers. In order to facilitate more effective follow-up, different prognostic models have been developed to calculate the individual risk of recurrence and to design an optimal follow-up strategy^216^. For patients with early-stage cervical cancer after surgical treatment including fertility sparing treatment, simple and radical hysterectomy, the Annual Recurrence Risk Model (ARRM) with on-line risk calculation was proposed to tailor the follow-up strategy^217^. The ARRM model allows to assess the risk of recurrence in each year after surgery for 5 prognostically different cohorts, but also the potential site of recurrence (pelvic vs distant site). The ARRM model consists of five prognostic variables from initial tumour diagnosis (i.e., maximal pathologic tumour diameter, tumour histotype, grade, number of positive pelvic LN and presence of LVSI) and helps stratifying patient follow-up per their risk profile. The model enables to differentiate between the lowest risk group with excellent prognosis where no regular follow-up is needed and highest risk group which will likely not benefit from any follow-up owing to prevailing distant metastases and expectedly very poor prognosis. The group with intermediate (26-50 points) and high-intermediate risks (51-75 points) has a risk of pelvic recurrence 5.2% and 13.7% for the first year of follow-up which steadily decreased by year three 3.2% for intermediate and by year five 3.9% for high-intermediate risk group. Both groups will benefit from tailored follow-up directed to pelvis using pelvic MRI or ultrasound 6 months after surgery and yearly for 3 to 5 years. The limitation of the model is that it is restricted to a group of early-stage disease treated by primary surgery and has not yet been prospectively externally validated. Similarly, prognostic factors were used to develop nomograms for 2-year progression-free survival, 5-year overall survival, and pelvic recurrence for locally advanced cervical cancer clinically limited to the pelvis treated with concurrent cisplatin-based chemotherapy and radiotherapy ^216^.

Cervical cancer survivors have an increased risk of developing a second malignancy compared to the general population^218^. This is particularly pronounced for cancer caused by smoking- and radiation, where the risk remained significantly elevated after ten years of follow-up. Radiation-induced second cancers, especially at radiated sites near the cervix include for example sigmoid colon, rectum/anus, urinary bladder. Counseling cervical cancer survivors about the risk of a second malignancies and active measures against smoking may become an important part of follow-up. Cancer survivors should participate in standard breast, colorectal, melanoma, lung screening programs according to national guidelines.

In case of suspected tumour persistence, recurrence or a second primary cancer, it is mandatory to confirm the finding by histological examination, if possible. For pelvic lesions, such as deeply located lesions in the endocervix (in case of conservative treatment or after definitive chemoradiotherapy), new lesions in the uterine cavity, involvement of parametrium or lymphnodes and others, ultrasound-guided tru-cut biopsy is the preferred method. In case of clinically or radiologically suspicious disease, a negative biopsy may not be conclusive. In case of inconclusive findings, repeat ultrasound-guided tru-cut biopsy with multiple cores taken from viable tissue (visualized on Doppler) is recommended. For any disease outside the pelvis, ultrasound-guided or CT-guided methods can be used to achieve pathological confirmation.

After follow-up at a specialized centre, when patients are referred back to their gynaecologist, long-term gynaecological follow-up is recommended as in the general population (annual population screening with physical and pelvic examination). A survivorship care plan consisting of a treatment summary, follow-up recommendations, and expedited referral procedures for suspected recurrence is suggested for a smooth transition from specialized cancer care to primary care.

**Quality of life and side-effects follow-up**

*General considerations*: in addition to follow-up for cancer recurrence, side effects of cancer and its treatment should be carefully prevented, detected, and monitored from the time of diagnosis, during and after treatment over the longterm. Acute (immediate or short-term) side effect develops right after exposure. Persistent (long-term) - side effects arise during treatment and may persist over time (months to years after treatment is completed). Late side effects (latent) first appear months to years after the end of treatment. There is no standard definition of the transition time between acute and persistent side effects, but immediate side effects usually subside within the first few months after treatment. Some late side effects of radiotherapy may occur more than 2-3 years after the end of treatment, justifying the need for long-term follow-up.

Cervical cancer treatment can cause immediate common side effects such as a tight and shorter vagina, pain during intercourse and menopausal symptoms. Side effects vary depending on the type of treatments. After cervical cancer surgery, patients may experience pain, difficulty urinating (retention symptoms) and defecating, and may gradually develop lymphedema. Patients treated with radiotherapy reported that bowel cramps, diarrhea and bladder irritation during treatments intensified in the first 3 weeks with a plateau at the 5^th^ week of treatment^219^^,^^220^. Skin irritation, nausea, fatigue may also occur. If treatment includes platinum-based chemotherapy, patients often report peripheral neuropathy at the end of treatment^219^^,^^220^. Regardless of treatment, patients suffer from poor quality of life (particularly in physical, social domains and well-being) and psychological distress.

Preventive supportive dietary measures, and care treatments such as loperamide, antispasmodic drugs, hydration counselling, should be considered when appropriate during radiation. Prevention of vaginal stenosis can start within one month after irradiation when acute mucositis reaction is resolving and it is carried out on a long-term basis^221^. The screening (follicle-stimulating hormone, luteinizing hormone, and estradiol) and treatment for premature ovarian failure is recommended if the treatment might impair gonadal hormone function.

Although quality of life and psychological distress improve within a year after treatment, patients often report persistent treatment-related side effects, including but not limited to menopausal symptoms, altered body image, sexual/vaginal dysfunction, diarrhea, lymphedema, peripheral neuropathy, fatigue, and social difficulties^220^^,^^222^. Lower urinary tract and less commonly bowel dysfunctions, and sexual health problems are some common persistent or long-term toxicities associated with surgery. While fibrosis-related symptoms are mainly reported after radiotherapy (bladder urgency, diarrhea, vaginal stenosis). Retrospective evidence shows no significant differences in oncological outcomes and overall rates of side effects between patients treated with radical hysterectomy and postoperative chemoradiotherapy for LN metastasis compared with the definitive chemoradiotherapy group^223^. However, both strategies are associated with different types of adverse events. Lower extremity lymphedema was more commonly reported after combined treatment with radical surgery and postoperative radiotherapy, whereas bladder or bowel problems and sexual dysfunction were more commonly reported after primary chemoradiotherapy. Lymphedema is one of the most frequent long-term side effects of surgery and/or radiation to the nodal group. Preventive recommendations include maintenance a normal body weight/avoiding weight gain in patients and a supervised exercise regimen. Compression (compressing arments or bandages) and physiotherapy should also be suggested. Treatment of lymphedema should be performed by a certified lymphedema specialist. Persistent fatigue is frequently reported after radiation and is associated with pre-existing comorbidities, severe pain, radiation doses, and other late persistent organ-related morbidities^224^. Providers should inform and educate on sexual and vaginal health because vaginal stenosis and vulvovaginal dryness may occur. Vaginal dilation should be initiated early and performed indefinitely, with concurrent vaginal lubricants and topical estrogen recommended. If the ovarian function is not preserved (e.g. ovaries are not transposed from the field of radiation), premenopausal patients are particularly at risk of developing early menopausal symptoms, including osteoporosis with higher risk of bone loss and insufficiency fractures^225^. Bone density status should be evaluated after treatment and should be monitored long-term. Dual-energy x-ray absorptiometry (DEXA, or DXA scan) is recommended immediately after treatment. Prevention (calcium supplementation if dietary calcium intake is insufficient to reach 1300 mg/day and vitamin D, weight-bearing exercise, healthy diet and smoking cessation). Treatment of osteoporosis should be the same as in the general population. Osteoporosis is treated with bisphosphonates or denosumab and vitamin D. Hormonal treatment can be considered to relieve menopausal (vasomotor) symptoms and to prevent menopause-related health risks, including osteoporosis. There is no evidence to contraindicate the use of systemic or topical menopausal hormone therapy in women with cervical cancer, as these tumours are not hormone dependent. The relative contraindications for menopausal hormone therapy in cancer survivors reflect those for the general population, including caution in survivors with coronary heart disease or hypertension, in those with increased genetic risk for cancers, and in current smokers, especially if over 35 years. Combination treatment with estrogen and progestin (for survivors with an intact uterus) or estrogens alone (for survivors without a uterus) should be initiated and continued until the average age of natural menopause (50 to 51 years)^226^. Extended use of menopausal hormone therapy may be considered on a case-by-case basis. In addition to systemic hormonal therapies, local (vaginal) estrogen therapy (rings, suppositories, creams) may be considered to manage genitourinary symptoms of menopause, including vulvovaginal dryness and dyspareunia, as well as urinary symptoms of urgency, dysuria, or recurrent urinary tract infection^227^. Recently, tissues elective estrogen complexes (TSECs) conjugated estrogens/bazedoxifene has been approved by the FDA for the treatment of menopausal symptoms in postmenopausal women^228^. Lifestyle interventions are effective in improving fatigue, some physical functions and leading to weight loss in survivors. Psycho-educational programs could improvemental health (mood disorders) and sexuality difficulties^229-231^. Programs of physical activity have been developed for various cancers and have shown health benefit, mitigating side effects and improving quality of life, but have not been developed for cervical cancer patients^232^^,^^233^.

If a late side effect occurs months or years after cancer treatment, a recurrence or a second primary tumour should be carefully ruled out. The type and treatment of a late side effect is no different from the treatment of a long-term side effect. Referral to specialized care long-term side effects clinics is encouraged.

*How to follow-up*:

- **Patient-reported outcomes (PROs):** evidence suggests that measuring PROs is important to complement physician-reported adverse events and to improve the accuracy of capturing symptomatic adverse events, such as those related to frequency, severity, and disruption of daily activities. Several randomized clinical trials have shown that routine self-reporting of treatment-related adverse events improves tolerability and quality of life, reduces the number of emergencies, and improves survival^234^. However, this approach has not yet been developed for patients with cervical cancer. There are no gold standard PROs tools for monitoring of patients with cervical cancer. Quality of life questionnaires designed for cervical cancer (FACT-CX or the EORTC-CX24) can be used. However, although these tools include different dimensions of quality of life, they do not perfectly capture the side effects of the treatments. Patient self-reported side effects questionnaires, including frequency, grading (degree of side effects), and interference with daily life (e.g., NCI PRO CTC AE or the EORTC library) could be designed to better capture patient unique treatment experiences. There is no standard for how and how often to monitor for side effects in cervical cancers, however, general oncology guidelines recommend early identification of side effects and supportive care needs with periodic reassessment during follow-up^235^. The development of digital support tools is an opportunity to incorporate side effects monitoring into routine.
- **Checklist of long-term concerns:** to help physicians better monitor the side effects of treatment, the Gynecologic Cancer Intergroup - Symptom benefit committee has established a minimum checklist of key long-term concerns for gynecologic cancer survivors, including prevention, diagnosis, and treatment of long-term treatment-induced side effects (i. e., lymphedema, neuropathy, urinary/digestive disorders, fatigue, chronic pain, osteoporosis, sexual and hormonal disorders, cognitive problems); other health concerns, particularly sleep disorders, emotional difficulties and social difficulties; secondary and tertiary prevention with a particular focus on cardiovascular disease with lifestyle counselling^212^.

**Quality of life and palliative care**

Early palliative care should be integrated with oncological treatments and offered to all patients diagnosed with advanced cervical cancer in order to manage symptoms and improve quality of life. A multidisciplinary approach should be incorporated into the care plan with the aim of providing specific treatment for symptoms. Common clinical situations requiring palliation in advanced cervical cancer include pain, lymphedema, malignant intestinal obstruction, vaginal bleeding, malodorous vaginal discharge, renal failure, fistulas, cachexia, fatigue, and psychosocial suffering. Early palliative care is essential in providing, not only symptom control, but comprehensive, holistic care to those faced with advanced cervical cancer^236-238^.

**Pain in cervical cancer**

Severe pelvic pain is common in advanced cervical cancer. Pelvic invasion often induces nerve compression or infiltration with neuropathic pain as a result (which is the most difficult to control). Apart from direct cancer injury, chemotherapy, radiotherapy, and surgery can also induce nerve damage^239^^,^^240^.

A variety of strong opioids are available for moderate to severe cancer-related pain and there is no superiority of one over another; however, the opioid of first choice for moderate to severe cancer pain is oral morphine^241^. Extended-release presentations, other opioids, or alternative routes (transdermic, subcutaneous) can be required in specific situations (i.e. intestinal obstruction, problems with swallowing, renal failure, toxicity). When there is a neuropathic pain component, opioids alone may not provide sufficient pain relief; in this case, the use of adequate doses of analgesic adjuvants is useful. Gabapentin, pregabalin, duloxetine and tricyclic antidepressants are strongly recommended as adjuvants and also as single agents for first-line treatment of neuropathic pain^241^.

Severe pelvic cancer pain unresponsive to an opioid regimen can benefit from other procedures like plexus block or spinal analgesia techniques^242^. Spinal analgesia techniques are expensive, with risk of permanent neurological injury, and require specialized equipment and well-trained staff members. These techniques can be considered for patients at the end of life when other methods are no longer useful, according to clinical condition and patient’s preferences^239^.

Palliative EBRT can be effective for painful pelvic progression (if no previous pelvic irradiation) and bone metastasis. In this situation of advanced non curable disease, hypofractionated palliative irradiation regimens are encouraged for to relief pain. Indication of palliative irradiation should take in count patient's prognosis, symptoms, performance status, ability to attend the hospital for treatment, etc^239^^,^^243^^,^^244^.

The malignant psoas syndrome is a rare and challenging cancer pain state with a symptomatic continuum of deep somatic nociceptive (muscle inflammation and spasm) and peripheral neuropathic pain (lumbar plexus injury). It is often refractory to standard multi-modal analgesic therapy and thus, eventually, needs advanced strategies for pain relief^245^.

**Lymphedema**

Malignant lymphedema of the lower limb significantly reduces the quality of life of patients by changing body image, causing pain, immobility, and fluid secretion. It is also associated with an increased risk of recurrent local infections (e.g., cellulitis, erysipelas). In the context of cervical cancer, lymphedema of the lower extremities often has multiple causes including surgical interventions (e.g., lymphadenectomy), irradiation, or tumour compression of lymphatic vessels^246^.

The evolution of treatments induced lymphedema is often chronic and generally cannot be cured. Surgical intervention is not indicated. The basic therapy consists of skin care, skin sanitation if needed, manual lymphatic drainage and compression therapy which in many cases should be realized by specialist professionals. In the case of spontaneous lymph fluid discharge targeted percutaneous lymphatic drainage can be effective^247^.

**Malignant intestinal obstruction**

The development of a malignant intestinal obstruction is common in patients with gynaecological pelvic cancers and is associated with symptoms like pain, nausea, vomiting, constipation, or paroxysmal diarrhea. Treatment should primarily be symptom oriented. Medication must be given via parenteral routes. The medical management of malignant intestinal obstruction consists of antisecretory, anti-inflammatory, antiemetic, pain relief and promoting emptying strategies and drugs^248^.

Nausea and vomiting can be controlled by antiemetics (e.g., metoclopramide), antipsychotics (e.g., olanzapine), corticosteroids (e.g., dexamethasone) or gastric protectors (e.g., ranitidine). The temporary placement of a nasogastric tube can be considered when the patient is experiencing discomfort (for example, continuous vomiting). On the other hand, gastrointestinal secretion inhibitors such as anticholinergics (e.g., butyl-scopolamine or octreotide; of the two, octreotide has shown itself to be superior to butyl-scopolamine) must be considered^249^.

Although malignant intestinal obstruction is associated with a poor prognosis, surgical interventions may be indicated in certain patients with good performance status and stable disease. Individualization of the decision made by a multidisciplinary team is extremely important; some parameters have been found to be related to a bad prognosis for the use of a surgical approach: ECOG, high serum urea and low albumin levels^250^.

Parenteral nutrition can be discussed for patients with good performance status and when it is expected that the obstruction can be solved. Otherwise, it would not make sense since it does not provide comfort and could be a source of greater morbidity. The usefulness and tolerability of parenteral fluid replacement and/or parenteral nutrition should be carefully discussed with the patient^251^. For patients at the end of life, parenteral nutrition has shown to be of limited use, as cachexia does not improve with it. Eating should be a pleasant experience for the patient during this period, not a source of stress. In cases where the medical team expects that obstruction can be improved with conservative or palliative surgery, parenteral nutrition for 7-10 days prior to the intervention can reduce postoperative infections, hospital stay, and postsurgical mortality. In such cases, it is a beneficial option for optimizing the patient for the surgery.

**Vaginal bleeding**

Bleeding is a frequent symptom in advanced cervical cancer and may be a cause of death (6%)^252^. The current approach depends on the available resources. Interventions for treatment of vaginal bleeding in women with advanced cervical cancer include tranexamic acid, vaginal packing (with or without formalin-soaked packs), interventional radiology (selective embolization) in addition to palliative radiotherapy (when it is feasible)^253^. There is no evidence from controlled trials supporting or refuting the use of any of the proposed interventions compared with radiotherapy. In the case of major bleeding, palliative sedation can be considered.

**Malodorous vaginal discharge**

Necrotic tumour often induces malodorous vaginal discharge that can be improved with vaginal washing and the use of a metronidazole tablet intravaginally^254^.

**Renal failure**

Renal failure induced by obstructive nephropathy is common among patients with compressive pelvic advanced tumour and may be the result of end-stage kidney disease. Symptoms such as dyspnea, pruritus, or delirium can appear and exacerbate other symptoms like edema and pain^239^^,^^255^.

Urinary derivation by ureteral stent or percutaneous nephrostomy could be a solution to hydronephrosis when the aim is to prevent the patient from dying from uremia and not from cervical cancer. There are no clear guidelines to exactly predict which patients will benefit from percutaneous nephrostomy in terms of survival and quality of life. A retrospective study suggested that percutaneous nephrostomy can be of clinical benefit for patients with recurrent cervical cancer and good performance status and may prolong survival. However, complications of percutaneous nephrostomy are frequent, including urinary tract infection (20%) and catheter loss (20%), pain induced by the catheter and most patients still die from renal failure. These data highlight the importance of carefully selecting patients who can derive benefit from this procedure^256^^,^^257^. Ascites, poor ECOG, diabetes mellitus, low serum albumin, hyponatremia, malignancy-related events and azotemia have been shown in different studies to result in a bad prognosis for percutaneous nephrostomy^258^^,^^259^.

When it is technically feasible, instead of percutaneous nephrostomy, a ureteral stent is an easier and less invasive procedure. In particular, tandem ureteral stenting has shown better results than a single ureteral stent^260^. In the case of urinary retention, urinary catheter is a good and easy palliative option to provide relief^254^.

**Fistula**

Rectal-vaginal and colon-bladder fistulas are common especially among patients with pelvic relapse after radiotherapy, which can lead to poor quality of life and psychological suffering. In these cases, surgery (colostomy or nephrostomy) can be discussed, but the benefit-risk ratio must be considered in each patient^240^.

**Cachexia and fatigue**

Cachexia and fatigue are two of the most common symptoms in advanced cervical cancer patients. Dedicated ESMO guidelines describe treatment and management for these symptoms^261^^,^^262^.

**Psychosocial suffering**

Cervical cancer may give rise to the social stigma associated with diseases of the genitals and a malodorous vaginal discharge, which may evoke feelings of humiliation, guilt, or shame. These feelings are related to suffering and loss of faith as well as being able to find meaning in life, which, along with the extreme physical symptoms produce a psychosocially complex environment that makes the multidisciplinary approach towards these patients and their families even more important^254^^,^^263^^,^^264^.

Physicians, nurses, psychologists, social workers, and community health workers can and must help to provide appropriate supportive counselling in a multidisciplinary approach. Psycho-oncologists and social workers collaborate with oncologists and palliative care providers to assess the severe, complex, or refractory psychological and social suffering of patients with cancer and their family members. Some standard simple self-reported questionnaires may be used to detect psychosocial distress such as the Distress Thermometer^240^^,^^265^. Non-pharmacologic interventions such as psychotherapy and supportive counselling are important approaches to help manage physical symptoms and improve quality of life.

Apart from the psychosocial circle, spiritual issues are extremely important at the end of life. Medical professionals should take care of this point and provide patients with the resources to access their needs, such as a spiritual guide^239^^,^^254^. In the case of clinically significant depression the use an antidepressant is indicated, especially fluoxetine^254^. Fluoxetine is one of the most commonly prescribed antidepressants and is proven to be safe, with a low risk of side effects and good tolerance. Its effectiveness in treating depression has been well-documented^254^.

**Cervical cancer in pregnancy**

Although the majority of cervical cancers coincidentally diagnosed in pregnancy are detected in early stages due to prenatal care, the association between cancer and pregnancy remains a significant challenge between optimal maternal therapy and fetal viability, and the decisions about therapy, taken by a multidisciplinary team, must be individualized. Many studies addressing the management of cervical cancer in pregnant women, mainly retrospective series, have been published in the course of the past 5 years, confirming the validity of the original ESGO/ESTRO/ESP guidelines^266-297^.

Imaging of pregnant cervical cancer patients as part of the primary work-up is still challenging. Radiologists play an important role in the multidisciplinary team in order to select the most optimal imaging strategies that balance maternal benefit with fetal risk and that are most likely to guide treatment decisions. Due to the absence of radiation exposure and highly accurate clinical performance, expert ultrasound and MRI remain the preferred imaging modalities. MRI has the added advantage of a more reproducible comprehensive organ or body region assessment, the ability of distant staging through whole-body evaluation, and the combination of anatomical and functional information by diffusion-weighted imaging which obviates the need for a gadolinium-based contrast-agent^298^. WB-DWI/MRI holds promise for accurate single-step staging with the absence or reduction of fetal radiation^299^. Because of limited experience and inherent radioactivity, PET-CT should be avoided during pregnancy.

Currently, there is no uniform standard for treatment. Several treatment modalities are available and should be discussed with the patient taking into account the tumour stage, gestational week of pregnancy and patient´s preferences. Depending on tumour stage and gestations age at diagnosis, delay of oncological treatment until fetal maturity (if possible >34 weeks of gestation) and initiate cancer-specific treatment immediately after delivery by cesarean section should be discussed. This option might be considered if the term or fetal maturity is approaching^300^.

Simple trachelectomy and PLND may be an option to be considered in a very select group of patients in order to preserve the pregnancy with the aim of definitive treatment at the time of delivery or shortly thereafter^284^^,^^288^. Abdominal radical trachelectomy may be discussed for selected patients with early-stage cervical cancer who want to preserve their pregnancy and who are not willing to expose the fetus to the risks associated with NACT^267^^,^^287^^,^^291^^,^^296^. Minimal invasive approach could be considered before 14-16 weeks of gestation^3^^,^^300^.

In locally advanced cervical cancer, platinum-based NACT may be offered to patients during the second and third trimesters and wishing to preserve an ongoing pregnancy in order to achieve fetal maturity, treat, stabilize and prevent further dissemination of the disease until the term, decrease the volume and extent of the tumour, and limit LN metastasis and distant micrometastasis during pregnancy^271^^,^^272^^,^^274^^,^^277^^,^^278^^,^^280^^,^^282^^,^^289^. However, long-term consequences of chemotherapy in the child are yet to be determined. Taxanes, when combined with platinum derivatives, may be safely administered in cervical cancer patients during the second and third trimesters of pregnancy, and thus could be an option^297^. Available data are inconsistent with respect to best regimen and maximal number of NACT cycles. Potential risks and benefits of cumulative chemotherapy have to be balanced with possible problems of fetal prematurity.

There are conflicting data with respect to mode of delivery. Occurrence of episiotomy scar recurrence can be associated with negative oncologic outcome. As spontaneous delivery appears to have negative prognostic impact and until more valid data available, cesarean section should be preferred mode of delivery.

If the women decide to not preserve the pregnancy, radical hysterectomy or definitive CTRT should be discussed according to the disease stage as recommended outside pregnancy. Pregnancy termination is recommended before any treatment after the first trimester, and fetus evaluation before CTRT. However, radical hysterectomy with fetus *in situ* is feasible without increased intra- or postoperative morbidity and does not worse oncologic outcome. Primary CTRT in early pregnancy induces spontaneous abortion and use of misoprostol can simplify uterine evacuation.

**Rare tumours**

This chapter was introduced to provide information on infrequent and uncommon tumour types of the uterine cervix. Due to their infrequency compared to particularly squamous cell carcinoma and adenocarcinoma, the title of “rare tumours” was selected. However, we are aware that this name could also be misleading to some point. The NCI defines rare tumours by an incidence of less than 15/100.000/year. The Joint Action on Rare Cancers of the European Union defines rare cancers those with a crude incidence rate of less than 6/100.000/year^301^. It is important to keep in mind that in many European countries the incidence of cervical carcinoma by itself is less than 15, which designates cervical carcinoma generally as a rare tumour. Nonetheless, identifying the rarest within the rare is important, due to the specific expertise needed to diagnose and treat patients affected by rare cervical cancers.

Rare cervical tumours represent a basket of tumours including a variety of epithelial, mesenchymal and other tumours and excluding squamous cell carcinoma and usual adenocarcinomas. It needs to be stressed that these tumours as a whole may affect patients of almost all age groups whereas the incidence of single tumour entities varies between the age groups.

Generally, the amount of scientific evidence for diagnosing and treating rare cervical tumours is limited, for the small number of cases reported. Most published articles are dealing with case reports or case series. Cervical neuroendocrine carcinoma, carcinosarcoma and sarcomas are the tumour types most frequently reported and discussed. In the recent WHO classification, carcinosarcoma is considered a type of carcinoma but it is not uncommon to find it discussed together with sarcomas.

In the National Cancer Database during the period 2004-2015, more than 100 000 new cases of cervical cancer were reported. Of these, squamous cell carcinoma accounted for about 76% and adenocarcinomas for about 23% of the cases^302^. Carcinosarcomas and sarcomas, including leiomyosarcomas, adenosarcomas and rhabdomyosarcomas accounted for most of the remaining approximately 1% of cases.

**Carcinosarcoma**

Carcinosarcomas seem to occur at older age and present at higher stage compared to squamous cell and adenocarcinomas^302^. The most common stage at presentation is FIGO IB and stage is the most important independent prognostic factor for recurrence and survival^303^. A heterologous component was found in about a third of the cases^303^. Lymph node involvement was found in a subset of cases but was not of prognostic value^303^. There is no standard therapy for cervical carcinosarcoma, but surgery followed by adjuvant radiotherapy with or without chemotherapy seems to improve overall and disease-free survival^303^. Prognosis seems to be better than for carcinosarcomas of the uterine corpus due to earlier symptoms and diagnosis^304^. Radical hysterectomy and bilateral salpingo-oophorectomy with pelvic lymphadenectomy and SN detection should be considered as the primary therapeutic modality^303^^,^^305^.

**Small cell neuroendocrine carcinoma**

The incidence of small cell neuroendocrine carcinoma of the cervix is not clear, even if quite a number of publications can be found in the literature^306^. It is likely that small cell neuroendocrine carcinoma accounts for about 2% of all cervical carcinomas. Most tumours are associated with HPV, mainly HPV types 16 and 18^307^. The incidence seems to rise during recent years maybe due to increased attention and improved diagnostic knowledge. Small cell carcinoma must be distinguished from poorly differentiated non-keratinizing squamous cell carcinoma and other mimics such as malignant lymphoma, rhabdomyosarcoma and undifferentiated carcinoma^308^. In a subset of cases patients may present with clinical and/or biochemical evidence of ectopic hormone production such as corticotropin, vasopressin, insulin, insulinoma-associated protein (INSM1), serotonin or parathormone and the related syndrome. A retrospective study on 93 small cell carcinomas of the cervix at stages I and II revealed invasion of lymphovascular spaces as a significant prognostic factor for both overall survival and disease-free survival and PLN metastasis and adjuvant chemotherapy with etoposide/cisplatin or irinotecan/cisplatin regimen as prognostic factors for disease-free survival^309^. A large study from China showed that advanced FIGO stage, large tumour size and older age were independent prognostic factor for overall survival whereas FIGO stage, tumour size and para-aortic LN metastasis were independent prognostic factors for progression-free survival^310^. Another large study from China revealed that LN status (p<0.01) and cancer directed surgery (p<0.01) were independent prognostic factors for FIGO I-IIA stage tumours whereas age (p<0.05), tumour size (p<0.01), chemotherapy (p<0.01) and radiation (p<0.01) were independent prognostic factors for FIGO stages IIB-IV^311^. In FIGO stages IB1-IIA1 treated with initial surgery, LN metastasis and resection margin involvement were poor prognostic factors of survival^312^. FIGO stage IVB seems to be exclusively associated with poor prognosis^313^. A meta-analysis showed FIGO staging, tumour size, parametrial involvement, resection margin, depth of stromal invasion, and LN metastasis as predictors of prognosis^314^. On the molecular level, neuroendocrine carcinoma of the cervix seems to be distinct from its counterpart in the lung and the urinary bladder by a significantly lower rate of coding mutations and TP53 mutations. In fact, PI3-kinase or MAPK pathway activating mutations were found in 67% of neuroendocrine carcinoma of the cervix. These findings suggest that on the molecular level neuroendocrine carcinomas of the cervix seem to be more similar to usual cervical carcinomas than to extra-cervical neuroendocrine carcinomas, particularly of lung and bladder^315^. Small cell neuroendocrine carcinomas are microsatellite stable but the data on PD-L1 expression are controversial with reported positivity in up to 50% of the cases^316^^,^^317^. Immunoreactivity for PARP-1 suggests a possible response to therapeutic PARP inhibition, but there is currently no information on a potential value of molecular alterations in this perspective, particularly of BRCA1/2 mutations or homologous recombination deficiency^316^. About a third of the cases seem to be immunoreactive for N-TRK but lack gene fusions^317^.

Evidence on treatment of neuroendocrine carcinoma is limited and standard therapy is lacking^318^^,^^319^. Radical surgery followed by chemotherapy may be a favorable alternative intervention for selected patients with advanced stage cancer^314^. NACT and adjuvant chemotherapy but not adjuvant radiotherapy seems to improve prognosis^320^. Paclitaxel plus cisplatin or paclitaxel plus carboplatin may be an alternative to etoposide/cisplatin, that remains the standard chemotherapy regimen used by most authors^306^^,^^314^.

Immune checkpoint inhibitor therapy is promising but lacks controlled evidence^318^. A multi-institutional retrospective analysis including 166 patients from 13 hospitals in Korea considered local radiotherapy as a definitive choice of treatment, particularly for advanced stage disease^321^. A systematic review including 11 studies with a total of 27 cases revealed that surgery after NACT may yield similar outcomes for locally advanced neuroendocrine carcinomas compared to CTRT^322^. The benefit of performing surgery as a primary approach could lie in the possibility of reserving CTRT for recurrences^322^. The need for effective systemic therapy is endorsed^321^.

**HPV-independent adenocarcinoma, clear cell type**

A retrospective study on clear cell carcinoma of the cervix includes 47 cases without exposure to diethylstilbestrol at a median age of 52 years^323^. About 50% percent of the cases presented at stage I. Almost 90% of the cases underwent radical hysterectomy and pelvic lymphadenectomy^323^. Within a multimodal approach, surgery seems to be effective in cases amenable to complete resection^323^. Advanced tumour stage, larger tumour size and PLN metastasis had negative effects on progression-free survival and overall survival. Adjuvant radiation therapy alone or concurrent CTRT after radical surgery did not affect survival in patients with risk factors^323^.

**Sarcomas**

Like carcinosarcomas, sarcomas seem to occur at older age and seem to present at higher stage compared to squamous cell and adenocarcinomas^302^. Leiomyosarcomas, adenosarcomas and rhabdomyosarcomas are the most frequently reported histological types, in addition a sarcoma NOS category has been reported^302^.

A meta-analysis on leiomyosarcomas (including 42 cases published in 29 articles) revealed age (</> 48 years) and mitotic count (</> 10/ 10 HPF) as independent prognostic factors for recurrence and age and performed hysterectomy as independent prognostic factors for survival^324^. Hysterectomy, without preference of radical hysterectomy, is considered the treatment of choice. Due to the rarity of this tumour, therapy mostly follows the treatment modalities for the more frequent uterine corpus counterpart^324^.

A single centre study on 49 adenosarcomas (19 from the cervix, 30 from the corpus) showed that disease-free survival was associated with tumour location, presence of a stalk connecting the tumour to cervix or corpus, heterologous elements and invasion of lymphovascular space^325^. In multivariate analysis, presence of tumour stalk remained an independently protective factor for recurrence and invasion of lymphovascular space a risk factor for recurrence^325^.

**References**

1.	Cibula, D.*, et al.* European Society of Gynaecological Oncology quality indicators for surgical treatment of cervical cancer. *Int J Gynecol Cancer* **30**, 3-14 (2020).

2.	Olawaiye, A.B., Baker, T.P., Washington, M.K. & Mutch, D.G. The new (Version 9) American Joint Committee on Cancer tumor, node, metastasis staging for cervical cancer. *CA Cancer J Clin* **71**, 287-298 (2021).

3.	Bhatla, N., Aoki, D., Sharma, D.N. & Sankaranarayanan, R. Cancer of the cervix uteri. *Int J Gynaecol Obstet* **143 Suppl 2**, 22-36 (2018).

4.	Bhatla, N. & Denny, L. FIGO Cancer Report 2018. *Int J Gynaecol Obstet* **143 Suppl 2**, 2-3 (2018).

5.	Corrigendum to "Revised FIGO staging for carcinoma of the cervix uteri" [Int J Gynecol Obstet 145(2019) 129-135]. *Int J Gynaecol Obstet* **147**, 279-280 (2019).

6.	Bhatla, N.*, et al.* Revised FIGO staging for carcinoma of the cervix uteri. *Int J Gynaecol Obstet* **145**, 129-135 (2019).

7.	Bhatla, N., Aoki, D., Sharma, D.N. & Sankaranarayanan, R. Cancer of the cervix uteri: 2021 update. *Int J Gynaecol Obstet* **155 Suppl 1**, 28-44 (2021).

8.	Sponholtz, S.E.*, et al.* From FIGO-2009 to FIGO-2018 in women with early-stage cervical cancer; Does the revised staging reflect risk groups? *Gynecologic oncology* **163**, 281-288 (2021).

9.	WHO Classification of Tumours, 5th edition, Volume 4: Female Genital Tumours. *https://*[*www.iarc.who.int/news-events/publication-of-the-who-classification-of-tumours-5th-edition-volume-4-female-genital-tumours/*](http://www.iarc.who.int/news-events/publication-of-the-who-classification-of-tumours-5th-edition-volume-4-female-genital-tumours/) (2020).

10.	Ronsini, C.*, et al.* The role of semiquantitative evaluation of lympho-vascular space invasion in early stage cervical cancer patients. *Gynecol Oncol* **162**, 299-307 (2021).

11.	Mills, A.M. PD-L1 Interpretation in Cervical Carcinomas: Proceedings of the ISGyP Companion Society Session at the 2020 USCAP Annual Meeting. *International journal of gynecological pathology : official journal of the International Society of Gynecological Pathologists* **40**, 1-4 (2021).

12.	Cibula, D.*, et al.* The European Society of Gynaecological Oncology/European Society for Radiotherapy and Oncology/European Society of Pathology Guidelines for the Management of Patients With Cervical Cancer. *International journal of gynecological cancer : official journal of the International Gynecological Cancer Society* **28**, 641-655 (2018).

13.	de Boer, P.*, et al.* Role of MRI in detecting involvement of the uterine internal os in uterine cervical cancer: systematic review of diagnostic test accuracy. *European journal of radiology* **82**, e422-428 (2013).

14.	Pradhan, T.S.*, et al.* Hydronephrosis as a prognostic indicator of survival in advanced cervix cancer. *International journal of gynecological cancer : official journal of the International Gynecological Cancer Society* **21**, 1091-1096 (2011).

15.	Nam, H.*, et al.* Prognostic significance of MRI-detected bladder muscle and/or serosal invasion in patients with cervical cancer treated with radiotherapy. *Br J Radiol* **83**, 868-873 (2010).

16.	Grigsby, P.W.*, et al.* FIGO 2018 staging criteria for cervical cancer: Impact on stage migration and survival. *Gynecol Oncol* **157**, 639-643 (2020).

17.	Matsuo, K., Machida, H., Mandelbaum, R.S., Konishi, I. & Mikami, M. Validation of the 2018 FIGO cervical cancer staging system. *Gynecol Oncol* **152**, 87-93 (2019).

18.	McComas, K.N.*, et al.* The variable impact of positive lymph nodes in cervical cancer: Implications of the new FIGO staging system. *Gynecol Oncol* **156**, 85-92 (2020).

19.	Matsuo, K.*, et al.* Significance of uterine corpus tumor invasion in early-stage cervical cancer. *Eur J Surg Oncol* **43**, 725-734 (2017).

20.	Narayan, K., Fisher, R. & Bernshaw, D. Significance of tumor volume and corpus uteri invasion in cervical cancer patients treated by radiotherapy. *International journal of gynecological cancer : official journal of the International Gynecological Cancer Society* **16**, 623-630 (2006).

21.	He, F.*, et al.* Influence of uterine corpus invasion on prognosis in stage IA2-IIB cervical cancer: A multicenter retrospective cohort study. *Gynecol Oncol* **158**, 273-281 (2020).

22.	Cibula, D.*, et al.* Tumour-free distance: a novel prognostic marker in patients with early-stage cervical cancer treated by primary surgery. *Br J Cancer* **124**, 1121-1129 (2021).

23.	Bizzarri, N.*, et al.* Validation of tumour-free distance as novel prognostic marker in early-stage cervical cancer: a retrospective, single-centre, cohort study. *Br J Cancer* **125**, 561-568 (2021).

24.	Quinn, B.A.*, et al.* Increasing age predicts poor cervical cancer prognosis with subsequent effect on treatment and overall survival. *Brachytherapy* **18**, 29-37 (2019).

25.	Chen, S., Tao, M., Zhao, L. & Zhang, X. The association between diabetes/hyperglycemia and the prognosis of cervical cancer patients: A systematic review and meta-analysis. *Medicine (Baltimore)* **96**, e7981 (2017).

26.	Cao, W.*, et al.* Prognostic role of pretreatment thrombocytosis on survival in patients with cervical cancer: a systematic review and meta-analysis. *World J Surg Oncol* **17**, 132 (2019).

27.	Koulis, T.A.*, et al.* Anemia, leukocytosis and thrombocytosis as prognostic factors in patients with cervical cancer treated with radical chemoradiotherapy: A retrospective cohort study. *Clin Transl Radiat Oncol* **4**, 51-56 (2017).

28.	Knoth, J.*, et al.* Clinical and imaging findings in cervical cancer and their impact on FIGO and TNM staging - An analysis from the EMBRACE study. *Gynecol Oncol* **159**, 136-141 (2020).

29.	Lindegaard, J.C., Petric, P., Lindegaard, A.M., Tanderup, K. & Fokdal, L.U. Evaluation of a New Prognostic Tumor Score in Locally Advanced Cervical Cancer Integrating Clinical Examination and Magnetic Resonance Imaging. *Int J Radiat Oncol Biol Phys* **106**, 754-763 (2020).

30.	Lindegaard, J.C.*, et al.* Prognostic implications of uterine cervical cancer regression during chemoradiation evaluated by the T-score in the multicenter XXX study. *International journal of radiation oncology, biology, physics* (2022).

31.	Cibula, D.*, et al.* Prognostic significance of low volume sentinel lymph node disease in early-stage cervical cancer. *Gynecologic oncology* **124**, 496-501 (2012).

32.	Pedone Anchora, L.*, et al.* Should the Number of Metastatic Pelvic Lymph Nodes be Integrated into the 2018 Figo Staging Classification of Early Stage Cervical Cancer? *Cancers (Basel)* **12**(2020).

33.	Peters, M.*, et al.* Risk factors for nodal failure after radiochemotherapy and image guided brachytherapy in locally advanced cervical cancer: An EMBRACE analysis. *Radiother Oncol* **163**, 150-158 (2021).

34.	Haldorsen, I.S., Lura, N., Blaakær, J., Fischerova, D. & Werner, H.M.J. What Is the Role of Imaging at Primary Diagnostic Work-Up in Uterine Cervical Cancer? *Current oncology reports* **21**, 77 (2019).

35.	Onal, C., Reyhan, M., Guler, O.C. & Yapar, A.F. Treatment outcomes of patients with cervical cancer with complete metabolic responses after definitive chemoradiotherapy. *Eur J Nucl Med Mol Imaging* **41**, 1336-1342 (2014).

36.	Onal, C., Guler, O.C., Reyhan, M. & Yapar, A.F. Long-term outcomes of cervical cancer patients with complete metabolic response after definitive chemoradiotherapy. *J Gynecol Oncol* **32**, e74 (2021).

37.	Beriwal, S.*, et al.* Complete metabolic response after definitive radiation therapy for cervical cancer: patterns and factors predicting for recurrence. *Gynecologic oncology* **127**, 303-306 (2012).

38.	Bollineni, V.R., Kramer, G., Liu, Y., Melidis, C. & deSouza, N.M. A literature review of the association between diffusion-weighted MRI derived apparent diffusion coefficient and tumour aggressiveness in pelvic cancer. *Cancer treatment reviews* **41**, 496-502 (2015).

39.	Gui, B.*, et al.* Prospective multimodal imaging assessment of locally advanced cervical cancer patients administered by chemoradiation followed by radical surgery-the "PRICE" study 2: role of conventional and DW-MRI. *Eur Radiol* **29**, 2045-2057 (2019).

40.	Thomeer, M.G.*, et al.* Evaluation of T2-W MR imaging and diffusion-weighted imaging for the early post-treatment local response assessment of patients treated conservatively for cervical cancer: a multicentre study. *Eur Radiol* **29**, 309-318 (2019).

41.	Testa, A.C.*, et al.* PRospective Imaging of CErvical cancer and neoadjuvant treatment (PRICE) study: role of ultrasound to predict partial response in locally advanced cervical cancer patients undergoing chemoradiation and radical surgery. *Ultrasound Obstet Gynecol* **51**, 684-695 (2018).

42.	Alsner, J., Overgaard, J., Tramm, T. & Lindegaard, J.C. Hypoxic gene expression is a prognostic factor for disease free survival in a cohort of locally advanced squamous cell cancer of the uterine cervix. *Acta oncologica (Stockholm, Sweden)* **61**, 172-178 (2022).

43.	Nicolás, I.*, et al.* HPV-negative tumors of the uterine cervix. *Mod Pathol* **32**, 1189-1196 (2019).

44.	Rodríguez-Carunchio, L.*, et al.* HPV-negative carcinoma of the uterine cervix: a distinct type of cervical cancer with poor prognosis. *BJOG : an international journal of obstetrics and gynaecology* **122**, 119-127 (2015).

45.	Lakomy, D.S.*, et al.* Immune correlates of therapy outcomes in women with cervical cancer treated with chemoradiotherapy: A systematic review. *Cancer Med* **10**, 4206-4220 (2021).

46.	Barrington, D.A., Riedinger, C., Haight, P.J., Tubbs, C. & Cohn, D.E. Pembrolizumab with or without bevacizumab for recurrent or metastatic cervical cancer: A cost-effectiveness analysis. *Gynecologic oncology* **165**, 500-505 (2022).

47.	Saglam, O. & Conejo-Garcia, J. PD-1/PD-L1 immune checkpoint inhibitors in advanced cervical cancer. *Integr Cancer Sci Ther* **5**(2018).

48.	Gu, X.*, et al.* Elevated PD-L1 expression predicts poor survival outcomes in patients with cervical cancer. *Cancer Cell Int* **19**, 146 (2019).

49.	Noh, J.J.*, et al.* Frequency of Mismatch Repair Deficiency/High Microsatellite Instability and Its Role as a Predictive Biomarker of response to Immune Checkpoint Inhibitors in Gynecologic Cancers. *Cancer Res Treat* (2021).

50.	Qu, X.*, et al.* Identification of a novel six-gene signature with potential prognostic and therapeutic value in cervical cancer. *Cancer Med* **10**, 6881-6896 (2021).

51.	Thomeer, M.G.*, et al.* Clinical examination versus magnetic resonance imaging in the pretreatment staging of cervical carcinoma: systematic review and meta-analysis. *Eur Radiol* **23**, 2005-2018 (2013).

52.	Epstein, E.*, et al.* Early-stage cervical cancer: tumor delineation by magnetic resonance imaging and ultrasound - a European multicenter trial. *Gynecologic oncology* **128**, 449-453 (2013).

53.	Alcazar, J.L.*, et al.* Magnetic resonance imaging and ultrasound for assessing parametrial infiltration in cervical cancer. A systematic review and meta-analysis. *Med Ultrason* **22**, 85-91 (2020).

54.	Pálsdóttir, K.*, et al.* Interobserver agreement of transvaginal ultrasound and magnetic resonance imaging in local staging of cervical cancer. *Ultrasound in obstetrics & gynecology : the official journal of the International Society of Ultrasound in Obstetrics and Gynecology* **58**, 773-779 (2021).

55.	Manganaro, L.*, et al.* Staging, recurrence and follow-up of uterine cervical cancer using MRI: Updated Guidelines of the European Society of Urogenital Radiology after revised FIGO staging 2018. *European radiology* **31**, 7802-7816 (2021).

56.	Sarabhai, T.*, et al.* Comparison of (18)F-FDG PET/MRI and MRI for pre-therapeutic tumor staging of patients with primary cancer of the uterine cervix. *Eur J Nucl Med Mol Imaging* **45**, 67-76 (2018).

57.	Nie, J.*, et al.* Diagnostic role of 18F-FDG PET/MRI in patients with gynecological malignancies of the pelvis: A systematic review and meta-analysis. *PLoS One* **12**, e0175401 (2017).

58.	Park, K.J.*, et al.* Carcinoma of the Cervix Histopathology Reporting Guide, 4th edition. International Collaboration on Cancer Reporting; Sydney, Australia. ISBN: 978-1-922324-24-5. (2021).

59.	Shim, S.H.*, et al.* Can simple trachelectomy or conization show comparable survival rate compared with radical trachelectomy in IA1 cervical cancer patients with lymphovascular space invasion who wish to save fertility? A systematic review and guideline recommendation. *PLoS One* **13**, e0189847 (2018).

60.	Feng, Y., Zhang, Z., Lou, T., Wang, S. & Bai, H. The safety of fertility preservation for microinvasive cervical adenocarcinoma: a meta-analysis and trial sequential analysis. *Arch Gynecol Obstet* **298**, 465-475 (2018).

61.	Baiocchi, G.*, et al.* Predictive Factors for Residual Disease After Conization in Cervical Cancer. *Ann Surg Oncol* **28**, 6673-6681 (2021).

62.	Bean, L.M., Ward, K.K., Plaxe, S.C. & McHale, M.T. Survival of women with microinvasive adenocarcinoma of the cervix is not improved by radical surgery. *Am J Obstet Gynecol* **217**, 332 e331-332 e336 (2017).

63.	Benoit, L.*, et al.* Does a pre-operative conization improve disease-free survival in early-stage cervical cancer? *Arch Gynecol Obstet* **303**, 231-239 (2021).

64.	Bogani, G.*, et al.* Primary conization overcomes the risk of developing local recurrence following laparoscopic radical hysterectomy in early stage cervical cancer. *Int J Gynaecol Obstet* **151**, 43-48 (2020).

65.	Buchanan, T., Pierce, J.Y., Graybill, W., Kohler, M. & Creasman, W. Why do we continue to overtreat stage Ia carcinoma of the cervix? *Am J Obstet Gynecol* **217**, 413-417 (2017).

66.	Demirkiran, F.*, et al.* Simple vaginal trachelectomy for early stage cervical cancer: A tertiary cancer center experience. *Ginekol Pol* **89**, 475-480 (2018).

67.	Du, Y. & Xu, Y. Less extensive surgery for patients with FIGO stage IA2 cervical cancer: A population-based study. *J Gynecol Obstet Hum Reprod* **51**, 102291 (2022).

68.	Escamilla Galindo, E.P.*, et al.* Post-Conization FIGO stage IA1 squamous cell cervical carcinoma; is hysterectomy necessary? *Eur J Obstet Gynecol Reprod Biol* **264**, 368-373 (2021).

69.	Hartman, C.A.*, et al.* Analysis of Conservative Surgical Treatment and Prognosis of Microinvasive Squamous Cell Carcinoma of the Cervix Stage IA1: Results of Follow-Up to 20 Years. *Int J Gynecol Cancer* **27**, 357-363 (2017).

70.	Hartman, C.A.*, et al.* Conservative treatment of microinvasive squamous cell carcinoma of the cervix stage IA1: Defining conization height to an optimal oncological outcome. *PLoS One* **16**, e0253998 (2021).

71.	Li, B., Shou, Y. & Zhu, H. Predictive value of hemoglobin, platelets, and D-dimer for the survival of patients with stage IA1 to IIA2 cervical cancer: a retrospective study. *J Int Med Res* **49**, 3000605211061008 (2021).

72.	Li, X., Xia, L., Chen, X., Fu, Y. & Wu, X. Simple conization and pelvic lymphadenectomy in early-stage cervical cancer: A retrospective analysis and review of the literature. *Gynecol Oncol* **158**, 231-235 (2020).

73.	Liu, Q.*, et al.* Simple Hysterectomy for Patients with Stage IA2 Cervical Cancer: A Retrospective Cohort Study. *Cancer Manag Res* **13**, 7823-7832 (2021).

74.	Lucchini, S.M.*, et al.* Conization and lymph node evaluation in low-risk cervical cancer. Is it time to avoid radical surgery? Retrospective series and literature review. *Eur J Obstet Gynecol Reprod Biol* **266**, 163-168 (2021).

75.	Machida, H.*, et al.* Characteristics and outcomes of reproductive-aged women with early-stage cervical cancer: trachelectomy vs hysterectomy. *Am J Obstet Gynecol* **219**, 461 e461-461 e418 (2018).

76.	MacNab, W.S.*, et al.* The Current Burden of Follow-up of Stage 1A1 Cervical Cancer. *J Low Genit Tract Dis* **21**, 268-271 (2017).

77.	Margolis, B.*, et al.* Prognostic significance of lymphovascular space invasion for stage IA1 and IA2 cervical cancer. *Int J Gynecol Cancer* **30**, 735-743 (2020).

78.	Matsuo, K.*, et al.* Risk of metachronous ovarian cancer after ovarian conservation in young women with stage I cervical cancer. *Am J Obstet Gynecol* **217**, 580 e581-580 e510 (2017).

79.	Nasioudis, D.*, et al.* Minimally invasive hysterectomy for stage IA cervical carcinoma: a survival analysis of the National Cancer Database. *Int J Gynecol Cancer* **31**, 1099-1103 (2021).

80.	Nica, A.*, et al.* Cervical conization and lymph node assessment for early stage low-risk cervical cancer. *Int J Gynecol Cancer* **31**, 447-451 (2021).

81.	Paul, C., Sharples, K.J., Baranyai, J., Jones, R.W. & Skegg, D.C. Outcomes for women without conventional treatment for stage 1A (microinvasive) carcinoma of the cervix. *Aust N Z J Obstet Gynaecol* **58**, 321-329 (2018).

82.	Pichlik, T.*, et al.* Individualization of surgical management of cervical cancer stages IA1, IA2. *Ceska Gynekol* **84**, 172-176 (2019).

83.	Plante, M., Renaud, M.C., Sebastianelli, A. & Gregoire, J. Simple Vaginal Trachelectomy: A Valuable Fertility-Preserving Option in Early-Stage Cervical Cancer. *Int J Gynecol Cancer* **27**, 1021-1027 (2017).

84.	Plante, M., Renaud, M.C., Sebastianelli, A. & Gregoire, J. Simple vaginal trachelectomy in women with early-stage low-risk cervical cancer who wish to preserve fertility: the new standard of care? *Int J Gynecol Cancer* **30**, 981-986 (2020).

85.	Ryckman, J.M., Lin, C., Simone, C.B., 2nd & Verma, V. Patterns of Care for Stage IA Cervical Cancer: Use of Definitive Radiation Therapy Versus Hysterectomy. *Int J Gynecol Cancer* **28**, 773-781 (2018).

86.	Wang, X., Bi, Y., Wu, H., Wu, M. & Li, L. Oncologic and obstetric outcomes after conization for adenocarcinoma in situ or stage IA1 cervical cancer. *Sci Rep* **10**, 19920 (2020).

87.	Yahata, H.*, et al.* Surgical treatment and outcome of early invasive adenocarcinoma of the uterine cervix (FIGO stage IA1). *Asia Pac J Clin Oncol* **14**, e50-e53 (2018).

88.	Devaja, O.*, et al.* Sentinel lymph node biopsy alone in the management of early cervical carcinoma. *Int J Gynecol Cancer* (2020).

89.	Park, K.J.*, et al.* Tumor Staging of Endocervical Adenocarcinoma: Recommendations From the International Society of Gynecological Pathologists. *Int J Gynecol Pathol* **40**, S92-S101 (2021).

90.	Teoh, D., Musa, F., Salani, R., Huh, W. & Jimenez, E. Diagnosis and Management of Adenocarcinoma in Situ: A Society of Gynecologic Oncology Evidence-Based Review and Recommendations. *Obstet Gynecol* **135**, 869-878 (2020).

91.	Ramirez, P.T.*, et al.* Minimally Invasive versus Abdominal Radical Hysterectomy for Cervical Cancer. *N Engl J Med* **379**, 1895-1904 (2018).

92.	Salvo, G.*, et al.* Open vs minimally invasive radical trachelectomy in early-stage cervical cancer: International Radical Trachelectomy Assessment Study. *Am J Obstet Gynecol* **226**, 97 e91-97 e16 (2022).

93.	Chacon, E.*, et al.* SUCCOR cone study: conization before radical hysterectomy. *Int J Gynecol Cancer* **32**, 117-124 (2022).

94.	Cibula, D.*, et al.* Sentinel lymph node mapping and intraoperative assessment in a prospective, international, multicentre, observational trial of patients with cervical cancer: The SENTIX trial. *Eur J Cancer* **137**, 69-80 (2020).

95.	Balaya, V.*, et al.* Long-term oncological safety of sentinel lymph node biopsy in early-stage cervical cancer: A post-hoc analysis of SENTICOL I and SENTICOL II cohorts. *Gynecol Oncol* **164**, 53-61 (2022).

96.	Lecuru, F.R.*, et al.* SENTICOL III: an international validation study of sentinel node biopsy in early cervical cancer. A GINECO, ENGOT, GCIG and multicenter study. *Int J Gynecol Cancer* **29**, 829-834 (2019).

97.	Cibula, D.*, et al.* A prospective multicenter trial on sentinel lymph node biopsy in patients with early-stage cervical cancer (SENTIX). *Int J Gynecol Cancer* **29**, 212-215 (2019).

98.	Tu, H.*, et al.* Sentinel lymph node biopsy versus pelvic lymphadenectomy in early-stage cervical cancer: a multi-center randomized trial (PHENIX/CSEM 010). *Int J Gynecol Cancer* **30**, 1829-1833 (2020).

99.	Cheng, H.*, et al.* Oncological Outcomes and Safety of Ovarian Preservation for Early Stage Adenocarcinoma of Cervix: A Systematic Review and Meta-Analysis. *Front Oncol* **9**, 777 (2019).

100.	Casey, L. & Singh, N. Metastases to the ovary arising from endometrial, cervical and fallopian tube cancer: recent advances. *Histopathology* **76**, 37-51 (2020).

101.	Zhou, L.*, et al.* Independent risk factors for ovarian metastases in stage IA-IIB cervical carcinoma. *Acta Obstet Gynecol Scand* **98**, 18-23 (2019).

102.	Cibula, D.*, et al.* Completion of radical hysterectomy does not improve survival of patients with cervical cancer and intraoperatively detected lymph node involvement: ABRAX international retrospective cohort study. *Eur J Cancer* **143**, 88-100 (2021).

103.	Escande, A.*, et al.* Outcome of early stage cervical cancer patients treated according to a radiosurgical approach: Clinical results and prognostic factors. *Gynecol Oncol* **144**, 541-546 (2017).

104.	Chargari, C.*, et al.* Radiotherapy of cervical cancer. *Cancer Radiother* **26**, 298-308 (2022).

105.	Vieira-Serna, S.*, et al.* Preoperative brachytherapy for early-stage cervical cancer: Systematic review and meta-analysis. *Gynecol Oncol* **169**, 4-11 (2022).

106.	Burbano, J.*, et al.* Neoadjuvant chemotherapy prior to fertility-sparing surgery in cervical tumors larger than 2 cm: a systematic review on fertility and oncologic outcomes. *Int J Gynecol Cancer* **31**, 387-398 (2021).

107.	Di Donato, V.*, et al.* Fertility-sparing surgery for women with stage I cervical cancer of 4 cm or larger: a systematic review. *J Gynecol Oncol* (2021).

108.	Feng, Y., Zhang, Z., Lou, T., Wang, S. & Bai, H. The security of radical trachelectomy in the treatment of IA-IIA cervical carcinoma requires further evaluation: updated meta-analysis and trial sequential analysis. *Arch Gynecol Obstet* **299**, 1525-1536 (2019).

109.	Kuznicki, M.L.*, et al.* Fertility-Sparing Surgery for Early-Stage Cervical Cancer: A Systematic Review of the Literature. *J Minim Invasive Gynecol* **28**, 513-526 e511 (2021).

110.	Nezhat, C., Roman, R.A., Rambhatla, A. & Nezhat, F. Reproductive and oncologic outcomes after fertility-sparing surgery for early stage cervical cancer: a systematic review. *Fertil Steril* **113**, 685-703 (2020).

111.	Pandraklakis, A.*, et al.* The Conundrum of Prematurity and Pregnancy Outcomes after Fertility Sparing Treatment Modalities for Early Stage Cervical Cancer: A Systematic Review of the Literature. *Folia Med (Plovdiv)* **62**, 453-461 (2020).

112.	Smith, E.S.*, et al.* Radical Trachelectomy for the Treatment of Early-Stage Cervical Cancer: A Systematic Review. *Obstet Gynecol* **136**, 533-542 (2020).

113.	van Kol, K.G.G., Vergeldt, T.F.M. & Bekkers, R.L.M. Abdominal radical trachelectomy versus chemotherapy followed by vaginal radical trachelectomy in stage 1B2 (FIGO 2018) cervical cancer. A systematic review on fertility and recurrence rates. *Gynecol Oncol* **155**, 515-521 (2019).

114.	Zhang, Q.*, et al.* Oncologic and obstetrical outcomes with fertility-sparing treatment of cervical cancer: a systematic review and meta-analysis. *Oncotarget* **8**, 46580-46592 (2017).

115.	Alvarez, R.M.*, et al.* MRI measurement of residual cervical length after radical trachelectomy for cervical cancer and the risk of adverse pregnancy outcomes: a blinded imaging analysis. *BJOG* **125**, 1726-1733 (2018).

116.	Ayhan, A.*, et al.* Oncological and obstetric outcomes after fertility-sparing radical abdominal trachelectomy for early stage cervical cancer: a tertiary centre's 10 years' experience. *J Obstet Gynaecol* **39**, 248-252 (2019).

117.	Balaya, V.*, et al.* Perioperative morbidity of radical trachelectomy with lymphadenectomy in early-stage cervical cancer: a French prospective multicentric cohort. *J Gynecol Oncol* **30**, e34 (2019).

118.	Bogani, G.*, et al.* Long-term results of fertility-sparing treatment for early-stage cervical cancer. *Gynecol Oncol* **154**, 89-94 (2019).

119.	Costales, A., Michener, C. & Escobar-Rodriguez, P.F. Radical Trachelectomy for Early Stage Cervical Cancer. *Curr Treat Options Oncol* **19**, 75 (2018).

120.	Cottrell, C.M., Ohaegbulam, G.C., Smith, J.R. & Del Priore, G. Fertility-sparing treatment in cervical cancer: Abdominal trachelectomy. *Best Pract Res Clin Obstet Gynaecol* **75**, 72-81 (2021).

121.	Cui, R.R.*, et al.* Trends in Use and Survival Associated With Fertility-Sparing Trachelectomy for Young Women With Early-Stage Cervical Cancer. *Obstet Gynecol* **131**, 1085-1094 (2018).

122.	Deng, X.*, et al.* Abdominal radical trachelectomy guided by sentinel lymph node biopsy for stage IB1 cervical cancer with tumors >2 cm. *Oncotarget* **8**, 3422-3429 (2017).

123.	Egashira, K.*, et al.* Infertility after abdominal trachelectomy. *Acta Obstet Gynecol Scand* **97**, 1358-1364 (2018).

124.	Fanfani, F.*, et al.* Oncologic and obstetric outcomes after simple conization for fertility-sparing surgery in FIGO 2018 stage IB1 cervical cancer. *Int J Gynecol Cancer* **31**, 452-456 (2021).

125.	Fokom Domgue, J. & Schmeler, K.M. Conservative management of cervical cancer: Current status and obstetrical implications. *Best Pract Res Clin Obstet Gynaecol* **55**, 79-92 (2019).

126.	Gabriele, V., Lecointre, L., Faller, E. & Akladios, C. Type B Laparoscopic Radical Trachelectomy with Uterine Artery Preservation for Stage IB1 Cervical Cancer. *J Minim Invasive Gynecol* **26**, 365 (2019).

127.	Gil-Ibanez, B.*, et al.* Vaginal fertility-sparing surgery and laparoscopic sentinel lymph node detection in early cervical cancer. Retrospective study with 15 years of follow-up. *Eur J Obstet Gynecol Reprod Biol* **251**, 23-27 (2020).

128.	Ind, T. Overview of fertility sparing treatments for cervical cancer. *Best Pract Res Clin Obstet Gynaecol* **75**, 2-9 (2021).

129.	Iwata, T.*, et al.* The validity of the subsequent pregnancy index score for fertility-sparing trachelectomy in early-stage cervical cancer. *Fertil Steril* **115**, 1250-1258 (2021).

130.	Kasius, J.C., van der Velden, J., Denswil, N.P., Tromp, J.M. & Mom, C.H. Neo-adjuvant chemotherapy in fertility-sparing cervical cancer treatment. *Best Pract Res Clin Obstet Gynaecol* **75**, 82-100 (2021).

131.	Kasuga, Y., Ikenoue, S., Tanaka, M. & Ochiai, D. Management of pregnancy after radical trachelectomy. *Gynecol Oncol* **162**, 220-225 (2021).

132.	Kohn, J.R., Katebi Kashi, P., Acosta-Torres, S., Beavis, A.L. & Christianson, M.S. Fertility-sparing Surgery for Patients with Cervical, Endometrial, and Ovarian Cancers. *J Minim Invasive Gynecol* **28**, 392-402 (2021).

133.	Laios, A.*, et al.* Ovarian transposition and cervical cancer. *Best Pract Res Clin Obstet Gynaecol* **75**, 37-53 (2021).

134.	Lee, C.Y.*, et al.* Outcome and Subsequent Pregnancy after Fertility-Sparing Surgery of Early-Stage Cervical Cancers. *Int J Environ Res Public Health* **17**(2020).

135.	Li, X.*, et al.* Oncological results and recurrent risk factors following abdominal radical trachelectomy: an updated series of 333 patients. *BJOG* **126**, 1169-1174 (2019).

136.	Li, X.*, et al.* Reproductive and obstetric outcomes after abdominal radical trachelectomy (ART) for patients with early-stage cervical cancers in Fudan, China. *Gynecol Oncol* **157**, 418-422 (2020).

137.	Machida, H.*, et al.* Fertility-sparing trachelectomy for early-stage cervical cancer: A proposal of an ideal candidate. *Gynecol Oncol* **156**, 341-348 (2020).

138.	Malmsten, C., Hellberg, P., Bergmark, K. & Dahm-Kahler, P. Long-term fertility, oncological, and quality-of-life outcomes after trachelectomy in early stage cervical cancer. *Arch Gynecol Obstet* **299**, 1033-1041 (2019).

139.	Martinelli, F.*, et al.* Conization and lymph node evaluation as a fertility-sparing treatment for early stage cervical cancer. *Int J Gynecol Cancer* **31**, 457-461 (2021).

140.	Matsuo, K.*, et al.* Trachelectomy for reproductive-aged women with early-stage cervical cancer: minimally invasive surgery versus laparotomy. *Am J Obstet Gynecol* **220**, 469 e461-469 e413 (2019).

141.	Matsuo, K.*, et al.* Ovarian Conservation and Overall Survival in Young Women With Early-Stage Cervical Cancer. *Obstet Gynecol* **129**, 139-151 (2017).

142.	Matsuo, K.*, et al.* Association between hospital surgical volume and perioperative outcomes of fertility-sparing trachelectomy for cervical cancer: A national study in the United States. *Gynecol Oncol* **157**, 173-180 (2020).

143.	Okugawa, K.*, et al.* Oncologic and obstetric outcomes and complications during pregnancy after fertility-sparing abdominal trachelectomy for cervical cancer: a retrospective review. *Int J Clin Oncol* **22**, 340-346 (2017).

144.	Okugawa, K.*, et al.* Evaluation of adjuvant chemotherapy after abdominal trachelectomy for cervical cancer: a single-institution experience. *Int J Clin Oncol* **26**, 216-224 (2021).

145.	Rendon, G.J.*, et al.* Oncological and obstetrical outcomes after neo-adjuvant chemotherapy followed by fertility-sparing surgery in patients with cervical cancer >/=2 cm. *Int J Gynecol Cancer* **31**, 462-467 (2021).

146.	Salvo, G.*, et al.* Open Versus Minimally Invasive Radical Trachelectomy in Early-Stage Cervical Cancer: International Radical Trachelectomy Assessment Study. *Am J Obstet Gynecol* (2021).

147.	Shah, J.S.*, et al.* Reproductive counseling and pregnancy outcomes after radical trachelectomy for early stage cervical cancer. *J Gynecol Oncol* **30**, e45 (2019).

148.	Simjak, P., Cibula, D., Parizek, A. & Slama, J. Management of pregnancy after fertility-sparing surgery for cervical cancer. *Acta Obstet Gynecol Scand* **99**, 830-838 (2020).

149.	Somigliana, E.*, et al.* Fertility preservation in women with cervical cancer. *Crit Rev Oncol Hematol* **154**, 103092 (2020).

150.	Tesfai, F.M.*, et al.* Fertility-sparing surgery of cervical cancer >2 cm (International Federation of Gynecology and Obstetrics 2009 stage IB1-IIA) after neoadjuvant chemotherapy. *Int J Gynecol Cancer* **30**, 115-121 (2020).

151.	Theplib, A., Hanprasertpong, J. & Leetanaporn, K. Safety and Prognostic Impacts of Ovarian Preservation during Radical Hysterectomy for Early-Stage Adenocarcinoma and Adenosquamous Cervical Cancer. *Biomed Res Int* **2020**, 5791381 (2020).

152.	Tsaousidis, C.*, et al.* Large Conization-Retrospective Monocentric Results for Fertility Preservation in Young Women with Early Stage Cervical Cancer. *Reprod Sci* **29**, 791-799 (2022).

153.	van der Plas, R.C.J., Bos, A.M.E., Jurgenliemk-Schulz, I.M., Gerestein, C.G. & Zweemer, R.P. Fertility-sparing surgery and fertility preservation in cervical cancer: The desire for parenthood, reproductive and obstetric outcomes. *Gynecol Oncol* (2021).

154.	Wu, C.J.*, et al.* Radical trachelectomy for early stage cervical cancer: A case series and literature review. *Taiwan J Obstet Gynecol* **56**, 143-146 (2017).

155.	Zusterzeel, P.L.M., Aarts, J.W.M., Pol, F.J.M., Ottevanger, P.B. & van Ham, M. Neoadjuvant Chemotherapy Followed by Vaginal Radical Trachelectomy as Fertility-Preserving Treatment for Patients with FIGO 2018 Stage 1B2 Cervical Cancer. *Oncologist* **25**, e1051-e1059 (2020).

156.	Smith, A.L.*, et al.* Conservative surgery in early-stage cervical cancer: what percentage of patients may be eligible for conization and lymphadenectomy? *Gynecol Oncol* **119**, 183-186 (2010).

157.	Morice, P.*, et al.* Oncologic results of fertility sparing surgery of cervical cancer: An updated systematic review. *Gynecol Oncol* **165**, 169-183 (2022).

158.	Batman, S.H. & Schmeler, K.M. Fertility-Sparing and Less Radical Surgery for Cervical Cancer. *Curr Oncol Rep* **24**, 1541-1548 (2022).

159.	Schaafsma, M., Plante, M., Mom, C.H. & van Trommel, N.E. Is less more in the surgical treatment of early-stage cervical cancer? *Curr Opin Oncol* **34**, 473-489 (2022).

160.	Schmeler, K.M.*, et al.* ConCerv: a prospective trial of conservative surgery for low-risk early-stage cervical cancer. *Int J Gynecol Cancer* **31**, 1317-1325 (2021).

161.	Slama, J.*, et al.* Analysis of risk factors for recurrence in cervical cancer patients after fertility-sparing treatment: The FERTIlity Sparing Surgery retrospective multicenter study. *Am J Obstet Gynecol* (2022).

162.	Bjorge, T., Skare, G.B., Bjorge, L., Trope, A. & Lonnberg, S. Adverse Pregnancy Outcomes After Treatment for Cervical Intraepithelial Neoplasia. *Obstet Gynecol* **128**, 1265-1273 (2016).

163.	Kyrgiou, M.*, et al.* Obstetric outcomes after conservative treatment for cervical intraepithelial lesions and early invasive disease. *Cochrane Database Syst Rev* **11**, CD012847 (2017).

164.	Kacerovsky, M.*, et al.* Cervical excisional treatment increases the risk of intra-amniotic infection in subsequent pregnancy complicated by preterm prelabor rupture of membranes. *Am J Obstet Gynecol* (2022).

165.	Ekdahl, L.*, et al.* A combination of second trimester oral metronidazole and no sexual intercourse during second and third trimester may reduce late miscarriage and premature delivery after fertility sparing radical trachelectomy. *Eur J Obstet Gynecol Reprod Biol* **265**, 90-95 (2021).

166.	Bentivegna, E.*, et al.* Oncological outcomes after fertility-sparing surgery for cervical cancer: a systematic review. *Lancet Oncol* **17**, e240-e253 (2016).

167.	Bentivegna, E.*, et al.* Fertility results and pregnancy outcomes after conservative treatment of cervical cancer: a systematic review of the literature. *Fertil Steril* **106**, 1195-1211 e1195 (2016).

168.	Luhrs, O., Ekdahl, L., Geppert, B., Lonnerfors, C. & Persson, J. Resection of the upper paracervical lymphovascular tissue should be an integral part of a pelvic sentinel lymph node algorithm in early stage cervical cancer. *Gynecol Oncol* (2021).

169.	Tran, A.Q.*, et al.* Robotic Radical Parametrectomy With Upper Vaginectomy and Pelvic Lymphadenectomy in Patients With Occult Cervical Carcinoma After Extrafascial Hysterectomy. *J Minim Invasive Gynecol* **24**, 757-763 (2017).

170.	Wang, Y.*, et al.* Is salvage radiotherapy optimal to patients with occult cervical cancer undergoing inadvertent simple hysterectomy? A propensity score-matched analysis of a nationwide clinical oncology database. *Jpn J Clin Oncol* **51**, 630-638 (2021).

171.	Potter, R.*, et al.* MRI-guided adaptive brachytherapy in locally advanced cervical cancer (EMBRACE-I): a multicentre prospective cohort study. *Lancet Oncol* **22**, 538-547 (2021).

172.	Potter, R.*, et al.* The EMBRACE II study: The outcome and prospect of two decades of evolution within the GEC-ESTRO GYN working group and the EMBRACE studies. *Clin Transl Radiat Oncol* **9**, 48-60 (2018).

173.	Lindegaard, J.C.*, et al.* Prognostic Implications of Uterine Cervical Cancer Regression During Chemoradiation Evaluated by the T-Score in the Multicenter EMBRACE I Study. *Int J Radiat Oncol Biol Phys* **113**, 379-389 (2022).

174.	Han, K., Milosevic, M., Fyles, A., Pintilie, M. & Viswanathan, A.N. Trends in the utilization of brachytherapy in cervical cancer in the United States. *Int J Radiat Oncol Biol Phys* **87**, 111-119 (2013).

175.	Schmid, M.P.*, et al.* Risk Factors for Local Failure Following Chemoradiation and Magnetic Resonance Image-Guided Brachytherapy in Locally Advanced Cervical Cancer: Results From the EMBRACE-I Study. *J Clin Oncol*, JCO2201096 (2023).

176.	Mazeron, R.*, et al.* 45 or 50 Gy, Which is the Optimal Radiotherapy Pelvic Dose in Locally Advanced Cervical Cancer in the Perspective of Reaching Magnetic Resonance Image-guided Adaptive Brachytherapy Planning Aims? *Clin Oncol (R Coll Radiol)* **28**, 171-177 (2016).

177.	Fenkell, L.*, et al.* Parametrial boost using midline shielding results in an unpredictable dose to tumor and organs at risk in combined external beam radiotherapy and brachytherapy for locally advanced cervical cancer. *Int J Radiat Oncol Biol Phys* **79**, 1572-1579 (2011).

178.	Lindegaard, J.C.*, et al.* Early clinical outcome of coverage probability based treatment planning for simultaneous integrated boost of nodes in locally advanced cervical cancer. *Acta Oncol* **56**, 1479-1486 (2017).

179.	Ramlov, A.*, et al.* Clinical implementation of coverage probability planning for nodal boosting in locally advanced cervical cancer. *Radiother Oncol* **123**, 158-163 (2017).

180.	Bacorro, W.*, et al.* Dose-volume effects in pathologic lymph nodes in locally advanced cervical cancer. *Gynecol Oncol* **148**, 461-467 (2018).

181.	Gosset, M.*, et al.* Should We Cease to Perform Salvage Hysterectomy After Chemoradiation and Brachytherapy in Locally Advanced Cervical Cancer? *Anticancer Res* **39**, 2919-2926 (2019).

182.	Kalash, R.*, et al.* Use of Functional Magnetic Resonance Imaging in Cervical Cancer Patients With Incomplete Response on Positron Emission Tomography/Computed Tomography After Image-Based High-Dose-Rate Brachytherapy. *Int J Radiat Oncol Biol Phys* **102**, 1008-1013 (2018).

183.	Bonlokke, S.*, et al.* The Diagnostic Value of Circulating Cell-Free HPV DNA in Plasma from Cervical Cancer Patients. *Cells* **11**(2022).

184.	da Costa, S.C.S.*, et al.* Neoadjuvant Chemotherapy With Cisplatin and Gemcitabine Followed by Chemoradiation Versus Chemoradiation for Locally Advanced Cervical Cancer: A Randomized Phase II Trial. *J Clin Oncol* **37**, 3124-3131 (2019).

185.	Wang, B., Tan, Y., Yang, X. & Man, X. Survival outcomes of neoadjuvant chemotherapy-related strategies compared with concurrent chemoradiotherapy for locally advanced cervical cancer: a meta-analysis of randomized controlled trials. *Arch Gynecol Obstet* **304**, 485-493 (2021).

186.	Duenas-Gonzalez, A.*, et al.* Phase III, open-label, randomized study comparing concurrent gemcitabine plus cisplatin and radiation followed by adjuvant gemcitabine and cisplatin versus concurrent cisplatin and radiation in patients with stage IIB to IVA carcinoma of the cervix. *J Clin Oncol* **29**, 1678-1685 (2011).

187.	Thomas, G. Are we making progress in curing advanced cervical cancer? *J Clin Oncol* **29**, 1654-1656 (2011).

188.	Horeweg, N.*, et al.* Adjuvant Systemic Therapy after Chemoradiation and Brachytherapy for Locally Advanced Cervical Cancer: A Systematic Review and Meta-Analysis. *Cancers (Basel)* **13**(2021).

189.	Tovanabutra, C.*, et al.* Long-Term Outcomes and Sites of Failure in Locally Advanced, Cervical Cancer Patients Treated by Concurrent Chemoradiation with or without Adjuvant Chemotherapy: ACTLACC Trial. *Asian Pac J Cancer Prev* **22**, 2977-2985 (2021).

190.	Mileshkin, L.R.*, et al.* Adjuvant chemotherapy following chemoradiation as primary treatment for locally advanced cervical cancer compared to chemoradiation alone: The randomized phase III OUTBACK Trial (ANZGOG 0902, RTOG 1174, NRG 0274). https://ascopubs.org/doi/abs/10.1200/JCO.2021.39.15_suppl.LBA3. (2021).

191.	Keys, H.M.*, et al.* Radiation therapy with and without extrafascial hysterectomy for bulky stage IB cervical carcinoma: a randomized trial of the Gynecologic Oncology Group. *Gynecol Oncol* **89**, 343-353 (2003).

192.	Mazeron, R.*, et al.* Post radiation hysterectomy in locally advanced cervical cancer: Outcomes and dosimetric impact. *Radiother Oncol* **120**, 460-466 (2016).

193.	Souza, E.C.A.*, et al.* Post-radiotherapy hysterectomy does not benefit females with cervical adenocarcinoma. *Mol Clin Oncol* **13**, 92 (2020).

194.	Pergialiotis, V.*, et al.* The impact of adjuvant hysterectomy on survival outcomes of patients with locally advanced cervical cancer: A network meta-analysis. *Eur J Surg Oncol* **48**, 261-267 (2022).

195.	Kokka, F.*, et al.* Hysterectomy with radiotherapy or chemotherapy or both for women with locally advanced cervical cancer. *Cochrane Database Syst Rev* **8**, CD010260 (2022).

196.	Miriyala, R., Mahantshetty, U., Maheshwari, A. & Gupta, S. Neoadjuvant chemotherapy followed by surgery in cervical cancer: past, present and future. *Int J Gynecol Cancer* **32**, 260-265 (2022).

197.	Gupta, S.*, et al.* Neoadjuvant Chemotherapy Followed by Radical Surgery Versus Concomitant Chemotherapy and Radiotherapy in Patients With Stage IB2, IIA, or IIB Squamous Cervical Cancer: A Randomized Controlled Trial. *J Clin Oncol* **36**, 1548-1555 (2018).

198.	Kenter, G.*, et al.* Results from neoadjuvant chemotherapy followed by surgery compared to chemoradiation for stage Ib2-IIb cervical cancer, EORTC 55994. https://ascopubs.org/doi/10.1200/JCO.2019.37.15_suppl.5503. (2019).

199.	Marchetti, C., Fagotti, A., Tombolini, V., Scambia, G. & De Felice, F. Survival and toxicity in neoadjuvant chemotherapy plus surgery versus definitive chemoradiotherapy for cervical cancer: A systematic review and meta-analysis. *Cancer Treat Rev* **83**, 101945 (2020).

200.	Morris, M.*, et al.* Pelvic radiation with concurrent chemotherapy compared with pelvic and para-aortic radiation for high-risk cervical cancer. *N Engl J Med* **340**, 1137-1143 (1999).

201.	Peters, W.A., 3rd*, et al.* Concurrent chemotherapy and pelvic radiation therapy compared with pelvic radiation therapy alone as adjuvant therapy after radical surgery in high-risk early-stage cancer of the cervix. *J Clin Oncol* **18**, 1606-1613 (2000).

202.	Landoni, F.*, et al.* Randomised study of radical surgery versus radiotherapy for stage Ib-IIa cervical cancer. *Lancet* **350**, 535-540 (1997).

203.	Cibula, D.*, et al.* Surgical treatment of "intermediate risk" lymph node negative cervical cancer patients without adjuvant radiotherapy-A retrospective cohort study and review of the literature. *Gynecol Oncol* **151**, 438-443 (2018).

204.	van der Velden, J.*, et al.* Analysis of isolated loco-regional recurrence rate in intermediate risk early cervical cancer after a type C2 radical hysterectomy without adjuvant radiotherapy. *Int J Gynecol Cancer* (2019).

205.	Nasioudis, D.*, et al.* Role of adjuvant radiation therapy after radical hysterectomy in patients with stage IB cervical carcinoma and intermediate risk factors. *Int J Gynecol Cancer* **31**, 829-834 (2021).

206.	Haesen, J.*, et al.* Radical hysterectomy without adjuvant radiotherapy in patients with cervix carcinoma FIGO 2009 IB1, with or without positive Sedlis criteria. *Gynecol Oncol* **162**, 539-545 (2021).

207.	Tewari, K.S.*, et al.* Bevacizumab for advanced cervical cancer: final overall survival and adverse event analysis of a randomised, controlled, open-label, phase 3 trial (Gynecologic Oncology Group 240). *Lancet* **390**, 1654-1663 (2017).

208.	Colombo, N.*, et al.* Pembrolizumab for Persistent, Recurrent, or Metastatic Cervical Cancer. *N Engl J Med* **385**, 1856-1867 (2021).

209.	Tewari, K.S.*, et al.* Survival with Cemiplimab in Recurrent Cervical Cancer. *N Engl J Med* **386**, 544-555 (2022).

210.	Perkins, V.*, et al.* Incorporation of whole pelvic radiation into treatment of stage IVB cervical cancer: A novel treatment strategy. *Gynecol Oncol* **156**, 100-106 (2020).

211.	Xu, J.Y.*, et al.* Local treatment improves survival in patients with stage IVB cervical cancer. *Gynecol Oncol* **165**, 538-545 (2022).

212.	Woopen, H.*, et al.* GCIG-Consensus guideline for Long-term survivorship in gynecologic Cancer: A position paper from the gynecologic cancer Intergroup (GCIG) symptom benefit committee. *Cancer Treat Rev* **107**, 102396 (2022).

213.	Elit, L.*, et al.* Follow-up for women after treatment for cervical cancer: a systematic review. *Gynecologic oncology* **114**, 528-535 (2009).

214.	Elit, L., Kennedy, E.B., Fyles, A. & Metser, U. Follow-up for cervical cancer: a Program in Evidence-Based Care systematic review and clinical practice guideline update. *Curr Oncol* **23**, 109-118 (2016).

215.	Song, D.*, et al.* The negative conversion of high-risk human papillomavirus and its performance in surveillance of cervical cancer after treatment: a retrospective study. *Arch Gynecol Obstet* **295**, 197-203 (2017).

216.	Rose, P.G.*, et al.* Nomograms Predicting Progression-Free Survival, Overall Survival, and Pelvic Recurrence in Locally Advanced Cervical Cancer Developed From an Analysis of Identifiable Prognostic Factors in Patients From NRG Oncology/Gynecologic Oncology Group Randomized Trials of Chemoradiotherapy. *Journal of clinical oncology : official journal of the American Society of Clinical Oncology* **33**, 2136-2142 (2015).

217.	Cibula, D.*, et al.* The annual recurrence risk model for tailored surveillance strategy in patients with cervical cancer. *European journal of cancer* **158**, 111-122 (2021).

218.	Arnold, M.*, et al.* Second primary cancers in survivors of cervical cancer in The Netherlands: Implications for prevention and surveillance. *Radiother Oncol* **111**, 374-381 (2014).

219.	Wiltink, L.M.*, et al.* A systematic review of the impact of contemporary treatment modalities for cervical cancer on women's self-reported health-related quality of life. *Support Care Cancer* **28**, 4627-4644 (2020).

220.	Lokich, E. Gynecologic Cancer Survivorship. *Obstet Gynecol Clin North Am* **46**, 165-178 (2019).

221.	Matos, S.R.L.*, et al.* Consensus for vaginal stenosis prevention in patients submitted to pelvic radiotherapy. *PloS one* **14**, e0221054 (2019).

222.	Plotti, F.*, et al.* Assessment of Quality of Life and Urinary and Sexual Function After Radical Hysterectomy in Long-Term Cervical Cancer Survivors. *Int J Gynecol Cancer* **28**, 818-823 (2018).

223.	Wenzel, H.H.B.*, et al.* Primary or adjuvant chemoradiotherapy for cervical cancer with intraoperative lymph node metastasis - A review. *Cancer treatment reviews* **102**, 102311 (2022).

224.	Smet, S.*, et al.* Risk Factors for Late Persistent Fatigue After Chemoradiotherapy in Patients With Locally Advanced Cervical Cancer: An Analysis From the EMBRACE-I Study. *Int J Radiat Oncol Biol Phys* **112**, 1177-1189 (2022).

225.	Salcedo, M.P.*, et al.* Pelvic fractures and changes in bone mineral density after radiotherapy for cervical, endometrial, and vaginal cancer: A prospective study of 239 women. *Cancer* **126**, 2607-2613 (2020).

226.	Type and timing of menopausal hormone therapy and breast cancer risk: individual participant meta-analysis of the worldwide epidemiological evidence. *Lancet* **394**, 1159-1168 (2019).

227.	Crean-Tate, K.K., Faubion, S.S., Pederson, H.J., Vencill, J.A. & Batur, P. Management of genitourinary syndrome of menopause in female cancer patients: a focus on vaginal hormonal therapy. *American journal of obstetrics and gynecology* **222**, 103-113 (2020).

228.	Kagan, R., Williams, R.S., Pan, K., Mirkin, S. & Pickar, J.H. A randomized, placebo- and active-controlled trial of bazedoxifene/conjugated estrogens for treatment of moderate to severe vulvar/vaginal atrophy in postmenopausal women. *Menopause* **17**, 281-289 (2010).

229.	Setyowibowo, H.*, et al.* Psychoeducation for breast cancer: A systematic review and meta-analysis. *Breast* **62**, 36-51 (2022).

230.	Chow, K.M., Chan, C.W., Chan, J.C., Choi, K.K. & Siu, K.Y. A feasibility study of a psychoeducational intervention program for gynecological cancer patients. *Eur J Oncol Nurs* **18**, 385-392 (2014).

231.	Chow, K.M.*, et al.* A theory-driven psycho-educational intervention programme for gynaecological cancer patients during treatment trajectory: A randomised controlled trial. *Psychooncology* **29**, 437-443 (2020).

232.	McGovern, A., Mahony, N., Mockler, D. & Fleming, N. Efficacy of resistance training during adjuvant chemotherapy and radiation therapy in cancer care: a systematic review and meta-analysis. *Support Care Cancer* **30**, 3701-3719 (2022).

233.	Yang, L.*, et al.* Effects of Exercise on Cancer Treatment Efficacy: A Systematic Review of Preclinical and Clinical Studies. *Cancer research* **81**, 4889-4895 (2021).

234.	Basch, E. New frontiers in patient-reported outcomes: adverse event reporting, comparative effectiveness, and quality assessment. *Annu Rev Med* **65**, 307-317 (2014).

235.	Banerjee, A.K.*, et al.* Patient-Reported Outcome Measures in Safety Event Reporting: PROSPER Consortium guidance. *Drug Saf* **36**, 1129-1149 (2013).

236.	Krakauer, E.L.*, et al.* Augmented Package of Palliative Care for Women With Cervical Cancer: Responding to Refractory Suffering. *JCO Glob Oncol* **7**, 886-895 (2021).

237.	Bercow, A.S.*, et al.* Palliative care referral patterns and measures of aggressive care at the end of life in patients with cervical cancer. *Int J Gynecol Cancer* **31**, 66-72 (2021).

238.	Brisson, M. & Drolet, M. Global elimination of cervical cancer as a public health problem. *Lancet Oncol* **20**, 319-321 (2019).

239.	Bates, M.J. & Mijoya, A. A review of patients with advanced cervical cancer presenting to palliative care services at Queen Elizabeth Central Hospital in Blantyre, Malawi. *Malawi Med J* **27**, 93-95 (2015).

240.	Cleary, J.F. Cervical Cancer: 90-70-90 and Palliative Care. *JCO Glob Oncol* **7**, 1426-1428 (2021).

241.	Fallon, M.*, et al.* Management of cancer pain in adult patients: ESMO Clinical Practice Guidelines. *Ann Oncol* **29**, iv166-iv191 (2018).

242.	Ertas, I.E.*, et al.* The effectiveness of subcutaneously implanted epidural ports for relief of severe pain in patients with advanced-stage gynecological cancer: a prospective study. *Agri* **26**, 8-14 (2014).

243.	Necessity of palliative care began right from the start of treatment in providing well being to patients of cervical cancer treated with radiotherapy. 2017. Cochrane Central Register of Controlled Trials (CENTRAL). https://[www.cochranelibrary.com/central/doi/10.1002/central/CN-01884320/full](http://www.cochranelibrary.com/central/doi/10.1002/central/CN-01884320/full). (2017).

244.	Hata, M.*, et al.* Radiation Therapy for Patients with Bone Metastasis from Uterine Cervical Cancer: Its Role and Optimal Radiation Regimen for Palliative Care. *Anticancer Res* **38**, 1033-1040 (2018).

245.	Stevens, M.J., Atkinson, C. & Broadbent, A.M. The malignant psoas syndrome revisited: case report, mechanisms, and current therapeutic options. *J Palliat Med* **13**, 211-216 (2010).

246.	Ridner, S.H., Doersam, J.K., Stolldorf, D.P. & Dietrich, M.S. Development and Validation of the Lymphedema Symptom Intensity and Distress Survey-Lower Limb. *Lymphat Res Biol* **16**, 538-546 (2018).

247.	Pottharst, A., Steckkönig, A. & Aulitzky, W. Subkutane Drainage zur Behandlung des malignen Lymphödems in der palliativen Situation nach Versagen der komplexen physikalischen Entstauungstherapie. *ZeitschriftfürPalliativmedizin* **10**, 51-54 (2009).

248.	Huang, X.*, et al.* Medical Management of Inoperable Malignant Bowel Obstruction. *Ann Pharmacother* **55**, 1134-1145 (2021).

249.	Davis, M.*, et al.* Medical management of malignant bowel obstruction in patients with advanced cancer: 2021 MASCC guideline update. *Support Care Cancer* **29**, 8089-8096 (2021).

250.	Bento, J.H.*, et al.* Surgical Management of Malignant Intestinal Obstruction: Outcome and Prognostic Factors. *Chirurgia (Bucur)* **114**, 343-351 (2019).

251.	Ripamonti, C. & Bruera, E. Palliative management of malignant bowel obstruction. *Int J Gynecol Cancer* **12**, 135-143 (2002).

252.	Kim, Y.J.*, et al.* Retrospective review of symptoms and palliative care interventions in women with advanced cervical cancer. *Gynecol Oncol* **139**, 553-558 (2015).

253.	Eleje, G.U., Eke, A.C., Igberase, G.O., Igwegbe, A.O. & Eleje, L.I. Palliative interventions for controlling vaginal bleeding in advanced cervical cancer. *Cochrane Database Syst Rev* **3**, CD011000 (2019).

254.	Krakauer, E.L.*, et al.* Essential Package of Palliative Care for Women With Cervical Cancer: Responding to the Suffering of a Highly Vulnerable Population. *JCO Glob Oncol* **7**, 873-885 (2021).

255.	Maguire, P.J., Sobota, A., Mulholland, D., Ryan, J.M. & Gleeson, N. Incidence, management, and sequelae of ureteric obstruction in women with cervical cancer. *Support Care Cancer* **28**, 725-730 (2020).

256.	Ramondetta, L. What is the appropriate approach to treating women with incurable cervical cancer? *J Natl Compr Canc Netw* **11**, 348-355 (2013).

257.	Pinto, C.S., Small, I.A., Ferreira, C.G. & Dienstmann, R. Palliative percutaneous nephrostomy in recurrent cervical cancer: Retrospective analysis of 50 consecutive cases. *J Clin Oncol* **25**, 19609 (2007).

258.	Perri, T.*, et al.* Palliative urinary diversion in patients with malignant ureteric obstruction due to gynaecological cancer. *BMJ Support Palliat Care* (2019).

259.	Prentice, J., Amer, T., Tasleem, A. & Aboumarzouk, O. Malignant ureteric obstruction decompression: how much gain for how much pain? A narrative review. *J R Soc Med* **111**, 125-135 (2018).

260.	Liu, K.L.*, et al.* Comparison of single and tandem ureteral stenting for malignant ureteral obstruction: a prospective study of 104 patients. *Eur Radiol* **29**, 628-635 (2019).

261.	Arends, J.*, et al.* Cancer cachexia in adult patients: ESMO Clinical Practice Guidelines(). *ESMO Open* **6**, 100092 (2021).

262.	Fabi, A.*, et al.* Cancer-related fatigue: ESMO Clinical Practice Guidelines for diagnosis and treatment. *Ann Oncol* **31**, 713-723 (2020).

263.	Mantegna, G.*, et al.* Long-term prospective longitudinal evaluation of emotional distress and quality of life in cervical cancer patients who remained disease-free 2-years from diagnosis. *BMC Cancer* **13**, 127 (2013).

264.	Ferrandina, G.*, et al.* Quality of life and emotional distress in early stage and locally advanced cervical cancer patients: a prospective, longitudinal study. *Gynecol Oncol* **124**, 389-394 (2012).

265.	Donovan, K.A., Grassi, L., McGinty, H.L. & Jacobsen, P.B. Validation of the distress thermometer worldwide: state of the science. *Psychooncology* **23**, 241-250 (2014).

266.	Beharee, N., Shi, Z., Wu, D. & Wang, J. Diagnosis and treatment of cervical cancer in pregnant women. *Cancer Med* **8**, 5425-5430 (2019).

267.	Douligeris, A., Prodromidou, A., Psomiadou, V., Iavazzo, C. & Vorgias, G. Abdominal radical trachelectomy during pregnancy: A systematic review of the literature. *J Gynecol Obstet Hum Reprod* **49**, 101607 (2020).

268.	Bigelow, C.A.*, et al.* Management and outcome of cervical cancer diagnosed in pregnancy. *Am J Obstet Gynecol* **216**, 276 e271-276 e276 (2017).

269.	Bourgioti, C., Konidari, M. & Moulopoulos, L.A. Imaging of Gynecologic Malignancy in a Reproductive Age Female: Cancer During Pregnancy. *Radiol Clin North Am* **58**, 413-430 (2020).

270.	De Vincenzo, R.*, et al.* Locally advanced cervical cancer complicating pregnancy: A case of competing risks from the Catholic University of the Sacred Heart in Rome. *Gynecol Oncol* **150**, 398-405 (2018).

271.	Guo, Y., Zhang, D., Li, Y. & Wang, Y. A case of successful maintained pregnancy after neoadjuvant chemotherapy plus radical surgery for stage IB3 cervical cancer diagnosed at 13 weeks. *BMC Pregnancy Childbirth* **20**, 202 (2020).

272.	Halaska, M.J., Drochytek, V., Shmakov, R.G. & Amant, F. Fertility sparing treatment in cervical cancer management in pregnancy. *Best Pract Res Clin Obstet Gynaecol* **75**, 101-112 (2021).

273.	Halaska, M.J.*, et al.* Characteristics of patients with cervical cancer during pregnancy: a multicenter matched cohort study. An initiative from the International Network on Cancer, Infertility and Pregnancy. *Int J Gynecol Cancer* (2019).

274.	Huang, H., Quan, Y., Qi, X. & Liu, P. Neoadjuvant chemotherapy with paclitaxel plus cisplatin before radical surgery for locally advanced cervical cancer during pregnancy: A case series and literature review. *Medicine (Baltimore)* **100**, e26845 (2021).

275.	Ishiguro, T.*, et al.* PET/MR imaging for the evaluation of cervical cancer during pregnancy. *BMC Pregnancy Childbirth* **21**, 288 (2021).

276.	Levy, L.*, et al.* Survival of the fetus: cervical cancer and pregnancy, a challenging combination. *Lancet* **396**, 725 (2020).

277.	Li, M.*, et al.* Management of Cervical Cancer in Pregnant Women: A Multi-Center Retrospective Study in China. *Front Med (Lausanne)* **7**, 538815 (2020).

278.	Lopez, A.*, et al.* Neoadjuvant chemotherapy in pregnant patients with cervical cancer: a Latin-American multicenter study. *Int J Gynecol Cancer* **31**, 468-474 (2021).

279.	Ma, J.*, et al.* Treatment and clinical outcomes of cervical cancer during pregnancy. *Ann Transl Med* **7**, 241 (2019).

280.	Mandic, A., Maricic, S., Malenkovic, G., Stojic, I. & Gutic, B. Neoadjuvant chemotherapy in locally advanced cervical cancer in pregnancy-Review of the literature. *J BUON* **25**, 597-604 (2020).

281.	Nocarova, L. & Ondrus, D. Cervical cancer in pregnancy. *Klin Onkol* **33**, 268-273 (2020).

282.	Oliveira, A.F., Souza, L., Paschoini, M.C., Murta, E.F.C. & Nomelini, R.S. Chemotherapy for cervical cancer in pregnancy. *J Obstet Gynaecol* **39**, 425-426 (2019).

283.	Perrone, A.M.*, et al.* Cervical cancer in pregnancy: Analysis of the literature and innovative approaches. *J Cell Physiol* **234**, 14975-14990 (2019).

284.	Perrotta, M.*, et al.* Simple trachelectomy with laparoscopic pelvic lymphadenectomy in a pregnant woman with a FIGO stage IA2 cervical cancer. *Int J Gynecol Cancer* **30**, 1652-1653 (2020).

285.	Puchar, A.*, et al.* Invasive and in situ cervical cancer associated with pregnancy: analysis from the French cancer network (CALG: Cancer Associe a La Grossesse). *Clin Transl Oncol* **22**, 2002-2008 (2020).

286.	Ramirez, P.T., Abu-Rustum, N.R. & Euscher, E. Conservative management of cervical cancer in pregnancy. *Int J Gynecol Cancer* **29**, 434-438 (2019).

287.	Rodolakis, A.*, et al.* Abdominal Radical Trachelectomy for Early-Stage Cervical Cancer During Pregnancy: A Provocative Surgical Approach. Overview of the Literature and a Single-Institute Experience. *Int J Gynecol Cancer* **28**, 1743-1750 (2018).

288.	Salvo, G., Frumovitz, M., Pareja, R., Lee, J. & Ramirez, P.T. Simple trachelectomy with pelvic lymphadenectomy as a viable treatment option in pregnant patients with stage IB1 (>/=2cm) cervical cancer: Bridging the gap to fetal viability. *Gynecol Oncol* **150**, 50-55 (2018).

289.	Song, Y., Liu, Y., Lin, M., Sheng, B. & Zhu, X. Efficacy of neoadjuvant platinum-based chemotherapy during the second and third trimester of pregnancy in women with cervical cancer: an updated systematic review and meta-analysis. *Drug Des Devel Ther* **13**, 79-102 (2019).

290.	Sonoda, K.*, et al.* Difficulty of cervical cancer diagnosis during pregnancy: A case series analysis of the clinicopathological characteristics and prognosis of cervical cancer diagnosed during pregnancy or within 6 months after parturition. *Mol Clin Oncol* **14**, 67 (2021).

291.	Stanca, M.*, et al.* The Double Life-Saving Approach of Abdominal Radical Trachelectomy during Pregnancy for Early-Stage Cervical Cancer-An Overview of the Literature and Our Institutional Experience. *J Pers Med* **11**(2021).

292.	Terukina, J., Takamatsu, M., Enomoto, T. & Baba, H. Anesthetic management of abdominal radical trachelectomy for uterine cervical cancer during pregnancy. *J Anesth* **31**, 467-471 (2017).

293.	Umemoto, M.*, et al.* Obstetrical prognosis of patients who underwent vaginal radical trachelectomy during pregnancy. *J Obstet Gynaecol Res* **45**, 1167-1172 (2019).

294.	Vasques, R.B.*, et al.* Evaluation of uterine cervical cancer in pregnancy: A cross-sectional study. *Eur J Obstet Gynecol Reprod Biol* **246**, 35-39 (2020).

295.	Wong, J.W.H., Sperling, M.M., Harvey, S.A., Killeen, J.L. & Carney, M.E. A fight-and-flight for life: A rare case of advanced cervical cancer in pregnancy. *Gynecol Oncol Rep* **32**, 100565 (2020).

296.	Yoshihara, K.*, et al.* The Safety and Effectiveness of Abdominal Radical Trachelectomy for Early-Stage Cervical Cancer During Pregnancy. *Int J Gynecol Cancer* **28**, 782-787 (2018).

297.	Zagouri, F.*, et al.* Taxanes during pregnancy in cervical cancer: A systematic review and pooled analysis. *Cancer Treat Rev* **79**, 101885 (2019).

298.	ACR Committee on Drugs and Contrast Media - Manual on Contrast Media. https://[www.acr.org/-/media/ACR/Files/Clinical-Resources/Contrast_Media.pdf](http://www.acr.org/-/media/ACR/Files/Clinical-Resources/Contrast_Media.pdf). (2022).

299.	Han, S.N.*, et al.* Feasibility of whole-body diffusion-weighted MRI for detection of primary tumour, nodal and distant metastases in women with cancer during pregnancy: a pilot study. *Eur Radiol* **28**, 1862-1874 (2018).

300.	Amant, F.*, et al.* Gynecologic cancers in pregnancy: guidelines based on a third international consensus meeting. *Ann Oncol* **30**, 1601-1612 (2019).

301.	Casali, P.G. & Trama, A. Rationale of the rare cancer list: a consensus paper from the Joint Action on Rare Cancers (JARC) of the European Union (EU). *ESMO Open* **5**(2020).

302.	Albert, A., Lee, A., Allbright, R. & Vijayakumar, S. Primary sarcoma of the cervix: an analysis of patient and tumor characteristics, treatment patterns, and outcomes. *J Gynecol Oncol* **31**, e25 (2020).

303.	Kimyon Comert, G.*, et al.* Therapy Modalities, Prognostic Factors, and Outcome of the Primary Cervical Carcinosarcoma: Meta-analysis of Extremely Rare Tumor of Cervix. *Int J Gynecol Cancer* **27**, 1957-1969 (2017).

304.	Clement, P.B., Zubovits, J.T., Young, R.H. & Scully, R.E. Malignant mullerian mixed tumors of the uterine cervix: a report of nine cases of a neoplasm with morphology often different from its counterpart in the corpus. *Int J Gynecol Pathol* **17**, 211-222 (1998).

305.	Sharma, N.K., Sorosky, J.I., Bender, D., Fletcher, M.S. & Sood, A.K. Malignant mixed mullerian tumor (MMMT) of the cervix. *Gynecol Oncol* **97**, 442-445 (2005).

306.	Gadducci, A., Carinelli, S. & Aletti, G. Neuroendrocrine tumors of the uterine cervix: A therapeutic challenge for gynecologic oncologists. *Gynecol Oncol* **144**, 637-646 (2017).

307.	Castle, P.E., Pierz, A. & Stoler, M.H. A systematic review and meta-analysis on the attribution of human papillomavirus (HPV) in neuroendocrine cancers of the cervix. *Gynecol Oncol* **148**, 422-429 (2018).

308.	Gardner, G.J., Reidy-Lagunes, D. & Gehrig, P.A. Neuroendocrine tumors of the gynecologic tract: A Society of Gynecologic Oncology (SGO) clinical document. *Gynecol Oncol* **122**, 190-198 (2011).

309.	Ishikawa, M.*, et al.* Prognostic factors and optimal therapy for stages I-II neuroendocrine carcinomas of the uterine cervix: A multi-center retrospective study. *Gynecol Oncol* **148**, 139-146 (2018).

310.	Chen, J.*, et al.* Prognostic factors and treatment of neuroendocrine tumors of the uterine cervix based on the FIGO 2018 staging system: a single-institution study of 172 patients. *PeerJ* **9**, e11563 (2021).

311.	Huang, R., Gan, Q. & Cheng, J. Prognostic Factors and Local Treatment Modalities of Small-Cell Carcinoma of the Cervix: An Analysis According to the International Federation of Gynecology and Obstetrics Stage. *Cancer Manag Res* **12**, 3445-3456 (2020).

312.	Kim, Y.M.*, et al.* Small cell carcinoma of the uterine cervix: clinicopathologic study of 20 cases in a single center. *Eur J Gynaecol Oncol* **30**, 539-542 (2009).

313.	Kawamura, M.*, et al.* The importance of choosing the right strategy to treat small cell carcinoma of the cervix: a comparative analysis of treatments. *BMC Cancer* **21**, 1046 (2021).

314.	Zhang, D. & Ma, X. Prognostic factors and outcomes of early-stage small cell neuroendocrine carcinoma of the cervix: 37 cases from a single center. *PeerJ* **7**, e6868 (2019).

315.	Hillman, R.T.*, et al.* Comparative genomics of high grade neuroendocrine carcinoma of the cervix. *PLoS One* **15**, e0234505 (2020).

316.	Carroll, M.R.*, et al.* Evaluation of PARP and PDL-1 as potential therapeutic targets for women with high-grade neuroendocrine carcinomas of the cervix. *Int J Gynecol Cancer* **30**, 1303-1307 (2020).

317.	Chen, L.*, et al.* PD-L1, Mismatch Repair Protein, and NTRK Immunohistochemical Expression in Cervical Small Cell Neuroendocrine Carcinoma. *Front Oncol* **11**, 752453 (2021).

318.	Tempfer, C.B.*, et al.* Neuroendocrine carcinoma of the cervix: a systematic review of the literature. *BMC Cancer* **18**, 530 (2018).

319.	Castelnau-Marchand, P.*, et al.* Multimodal Management of Locally Advanced Neuroendocrine Cervical Carcinoma: A Single Institution Experience. *Int J Gynecol Cancer* **28**, 1013-1019 (2018).

320.	Zhang, Y.*, et al.* Therapeutic strategy analysis of patients with advanced stage high-grade neuroendocrine cervical cancer: A real-world multicenter study. *Int J Gynaecol Obstet* (2022).

321.	Cho, W.K.*, et al.* Optimal treatment strategies for small cell carcinoma of the uterine cervix: A retrospective multi-center study (KROG 19-03). *Eur J Obstet Gynecol Reprod Biol* **258**, 396-400 (2021).

322.	Caruso, G.*, et al.* Radical Surgery After Neoadjuvant Chemotherapy for Locally Advanced Neuroendocrine Cancer of the Cervix. *Anticancer Res* **41**, 4431-4438 (2021).

323.	Yang, L.*, et al.* Clear Cell Carcinoma of the Uterine Cervix: A Clinical and Pathological Analysis of 47 Patients Without Intrauterine Diethylstilbestrol Exposure. *Int J Gynecol Cancer* **27**, 1009-1014 (2017).

324.	Kilic, C.*, et al.* Primary leiomyosarcoma of the uterine cervix: report of 4 cases, systematic review, and meta-analysis. *Tumori* **106**, 413-423 (2020).

325.	Yuan, Z.*, et al.* Uterine Adenosarcoma: A Retrospective 12-Year Single-Center Study. *Front Oncol* **9**, 237 (2019).
